# A novel viral strategy for host factor recruitment: The co-opted proteasomal Rpn11 protein interaction hub in cooperation with subverted actin filaments are targeted to deliver cytosolic host factors for viral replication

**DOI:** 10.1371/journal.ppat.1009680

**Published:** 2021-06-23

**Authors:** Melissa Molho, Wenwu Lin, Peter D. Nagy

**Affiliations:** Department of Plant Pathology, University of Kentucky, Lexington, Kentucky, United States of America; Agriculture and Agri-Food Canada, CANADA

## Abstract

Positive-strand (+)RNA viruses take advantage of the host cells by subverting a long list of host protein factors and transport vesicles and cellular organelles to build membranous viral replication organelles (VROs) that support robust RNA replication. How RNA viruses accomplish major recruitment tasks of a large number of cellular proteins are intensively studied. In case of tomato bushy stunt virus (TBSV), a single viral replication protein, named p33, carries out most of the recruitment duties. Yet, it is currently unknown how the viral p33 replication protein, which is membrane associated, is capable of the rapid and efficient recruitment of numerous cytosolic host proteins to facilitate the formation of large VROs. In this paper, we show that, TBSV p33 molecules do not recruit each cytosolic host factor one-by-one into VROs, but p33 targets a cytosolic protein interaction hub, namely Rpn11, which interacts with numerous other cytosolic proteins. The highly conserved Rpn11, called POH1 in humans, is the metalloprotease subunit of the proteasome, which couples deubiquitination and degradation of proteasome substrates. However, TBSV takes advantage of a noncanonical function of Rpn11 by exploiting Rpn11’s interaction with highly abundant cytosolic proteins and the actin network. We provide supporting evidence that the co-opted Rpn11 in coordination with the subverted actin network is used for delivering cytosolic proteins, such as glycolytic and fermentation enzymes, which are readily subverted into VROs to produce ATP locally in support of VRO formation, viral replicase complex assembly and viral RNA replication. Using several approaches, including knockdown of Rpn11 level, sequestering Rpn11 from the cytosol into the nucleus in plants or temperature-sensitive mutation in Rpn11 in yeast, we show the inhibition of recruitment of glycolytic and fermentation enzymes into VROs. The Rpn11-assisted recruitment of the cytosolic enzymes by p33, however, also requires the combined and coordinated role of the subverted actin network. Accordingly, stabilization of the actin filaments by expression of the *Legionella* VipA effector in yeast and plant, or via a mutation of *ACT1* in yeast resulted in more efficient and rapid recruitment of Rpn11 and the selected glycolytic and fermentation enzymes into VROs. On the contrary, destruction of the actin filaments via expression of the *Legionella* RavK effector led to poor recruitment of Rpn11 and glycolytic and fermentation enzymes. Finally, we confirmed the key roles of Rpn11 and the actin filaments *in situ* ATP production within TBSV VROs via using a FRET-based ATP-biosensor. The novel emerging theme is that TBSV targets Rpn11 cytosolic protein interaction hub driven by the p33 replication protein and aided by the subverted actin filaments to deliver several co-opted cytosolic pro-viral factors for robust replication within VROs.

## Author summary

(+)RNA viruses have to co-opt numerous host proteins to support their replication in infected cells. These viruses induce the biogenesis of viral replication organelles (VROs), the sites of replication, in cells. However, what the mechanism of subversion for most of the cytosolic host proteins is not yet dissected. In this paper, the authors used a plant (+)RNA virus, tomato bushy stunt virus (TBSV), to study the role of a cellular proteasomal protein, called Rpn11 (POH1), as a protein interaction hub. They show that knockdown of Rpn11 or retargeting Rpn11 into the nucleus and destruction of actin filaments diminishes TBSV replication in yeast and plant cells. This effect is due to diminished recruitment of pro-viral metabolic enzymes into VROs. Overall, data presented support a novel viral recruitment strategy for cytosolic host factors. Via the small viral replication protein, TBSV targets the cytosolic proteasomal Rpn11 protein interaction hub protein and the co-opted and stabilized actin filaments. The combined and coordinated subversion of Rpn11 and the actin network allows tombusviruses to gain access to abundant cytosolic proteins, such as the glycolytic and fermentation enzymes, which are then efficiently delivered to perform pro-viral functions within the VROs.

## Introduction

Positive-strand (+)RNA viruses code for only a small number of genes, therefore, they rely on subverting a long list of host factors to build robust viral replication organelles (VROs) or replication compartments [1–8]. Recent works with a (+)RNA virus, namely tomato bushy stunt virus (TBSV), opened up new frontiers on how viruses could force the host cells into facilitating the biogenesis of VROs, which consist of aggregated peroxisomal and ER membranes [9–11].

In addition to membranous compartments and transport vesicles, TBSV also hijacks several cytosolic host factors, such as the heat shock protein 70 (Hsp70), Vps4 and other ESCRT (the endosomal sorting complex required for transport) proteins, translation elongation factors, and a few DEAD-box RNA helicases [10, 12–18]. Moreover, TBSV recruits and compartmentalizes the cytosolic glycolytic and fermentation enzymes within the VROs for continuous ATP synthesis locally [19–21]. These co-opted host factors are required to assemble the VROs and to support robust viral RNA synthesis.

TBSV is especially useful in studying viral RNA replication based on the development of various unique *in vitro* and *in vivo* approaches including the use of yeast (*Saccharomyces cerevisiae*) model host [5, 22–29]. Direct translation of the single genomic (g)RNA of tombusviruses results in two replication proteins, termed p33 and p92^pol^. The abundant p33 RNA chaperone functions in recruitment of viral RNA template for replication and in the assembly of the membrane-bound viral replicase complexes (VRCs) [15, 30–34]. p92^pol^ is the RNA-dependent RNA polymerase (RdRp) [33, 35, 36], which is produced through translational readthrough of the p33 stop codon [37–39]. Both replication proteins are essential components of the tombusvirus VRCs [16, 36].

Tombusviruses co-opt many cytosolic host proteins into VROs via unknown mechanisms [8, 40]. It would take a large number of p33 molecules, which are membrane associated, to be involved in the rapid and efficient recruitment of all these cytosolic proteins one-by-one to facilitate the formation of large VROs. It is likely that TBSV p33 molecules will need help accomplishing such major tasks. A possible way for p33 to do the major recruitment task of the numerous host cytosolic proteins into VROs is to target putative cytosolic “protein interaction hub” proteins, which associate with many other cytosolic proteins. Among the putative cytosolic hub proteins known to interact with p33 replication protein is the Rpn11 proteasomal protein, which was originally identified in a systematic screen with TBSV based on a temperature-sensitive library of yeast mutants [41, 42]. The highly conserved Rpn11 (Regulatory Particle Non-ATPase, called *POH1* or *PSMD14* in humans) metalloprotease is part of the 19S regulatory particle, which constitutes the 26S proteasome lid [43]. Rpn11 essential function is to couple deubiquitination and degradation of proteasome substrates. In the presence of mutated Rpn11, polyubiquitinated proteins accumulate in yeast [44]. Proteasomes get degraded in the absence of Rpn11, making it essential for maintaining cellular protein homeostasis. An important function of Rpn11 is the formation of proteasome storage granules under certain cellular conditions, such quiescent stage in yeast [45]. However, Rpn11 is a major contributor to several pathways that are independent of its catalytic activity [44, 46]. The N-terminal part of Rpn11 contains the deubiquitinase (DUB, the catalytically active JAMM/MPN+) domain, whereas the C-terminal domain regulates the stability of the proteasomal lid, cell-cycle progression and mitochondrial fission and peroxisomal division [46, 47]. Thus, mutations in the multifunctional Rpn11 might have pleiotropic effects on the cell and its essential role makes it more challenging to dissect its pro-viral function in viral replication. Importantly, previous works with TBSV revealed that the canonical function of Rpn11 in the proteasome is not required for its pro-viral function [42]. Rpn11 is recruited into the VROs and it is important in facilitating the subversion of the pro-viral cytosolic DDX3-like Ded1 RNA helicase (called RH20 in plants) [42].

Because Rpn11 is a major protein interaction hub in cells and it is known to interact with the actin network [48, 49], in this work we explored the possible function of Rpn11 to facilitate the subversion of other cellular proteins into the TBSV VROs. This proposed function of Rpn11 may depend on the actin filaments, which are known to participate in the formation of TBSV VROs in yeast and plant cells [50]. The actin filaments are stabilized by TBSV via p33-based blocking of Cof1 (cofilin, also called actin depolymerization factor) function in disassembling actin filaments. The p33-mediated stabilized actin filaments then are used by TBSV to deliver vesicle cargoes, such as Rab5-decorated early endosomes and retromer tubular carriers into VROs to provide lipids/membranes and lipid enzymes for the biogenesis of VROs [51, 52]. In this work we provide supporting evidence that the stabilized actin network is used by TBSV for delivering cytosolic proteins, such as glycolytic and fermentation enzymes needed for local ATP generation into the large VROs. Altogether, we propose that p33 replication protein does not deliver each subverted cytosolic host factor one-by-one into VROs. Instead, TBSV targets Rpn11, which then serves as a major cytosolic protein interaction hub by facilitating the recruitment of other cellular cytosolic factors with the contribution of subverted actin filaments into the VROs. Therefore, the current work shows that Rpn11 is not only acting as a “matchmaker” between the viral p92^pol^ and the co-opted cellular DDX3-like Ded1p (RH20 in plants) DEAD-box helicase [42]. The emerging model is that TBSV targets Rpn11 cytosolic protein interaction hub driven by the p33 replication protein and aided by the stabilized and subverted actin filaments to deliver several co-opted cytosolic pro-viral factors for robust replication within VROs.

## Results

### Critical role of the cytosolic Rpn11 in assisting tombusviruses during recruitment of pro-viral glycolytic and fermentation enzymes into VROs

To test the concept that the subversion of several different cytosolic proteins by TBSV into the VROs might be facilitated by p33 replication protein via targeting a putative “cellular cytosolic protein interaction hub”, we decided to decipher the pro-viral function of Rpn11 proteasomal deubiquinase factor in subversion of other host factors. Because Rpn11 physically interacts with a large number (~1,000) of yeast proteins [49, 53], we decided to focus on the possible connection between Rpn11 and the host cytosolic glycolytic and fermentation enzymes, which are known to interact with Rpn11. The host cytosolic glycolytic and fermentation enzymes are readily subverted into VROs via p33 to produce ATP locally in support of VRO formation, VRCs assembly and viral RNA replication [19–21, 54]. However, the recruitment mechanism/pathway of various cytosolic proteins by a membrane-bound p33 into VROs is currently unknown.

Rpn11 is an essential protein, and therefore, we applied different approaches to manipulate Rpn11 availability for pro-viral functions. First, we knocked down Rpn11 mRNA level via VIGS in *N*. *benthamiana* (S1A Fig) followed by transient expression of p33 replication protein and three glycolytic enzymes and two fermentation enzymes, which are known pro-viral host factors [19–21, 55]. The glycolytic enzymes included the ATP generating Pgk1 (phosphoglycerate kinase 1) and PK (pyruvate kinase, Cdc19 in yeast and PKM2/PKLR in humans) as well as Fba2 (fructose 1,6-bisphosphate aldolase), whereas the fermentation enzymes included Pdc1 (pyruvate decarboxylase 1) and Adh1 (alcohol dehydrogenase 1). These fermentation enzymes are required for the replenishing of NAD^+^, which is critical regulatory compound in sustaining aerobic glycolysis pathway [56, 57].

**Fig 1:**
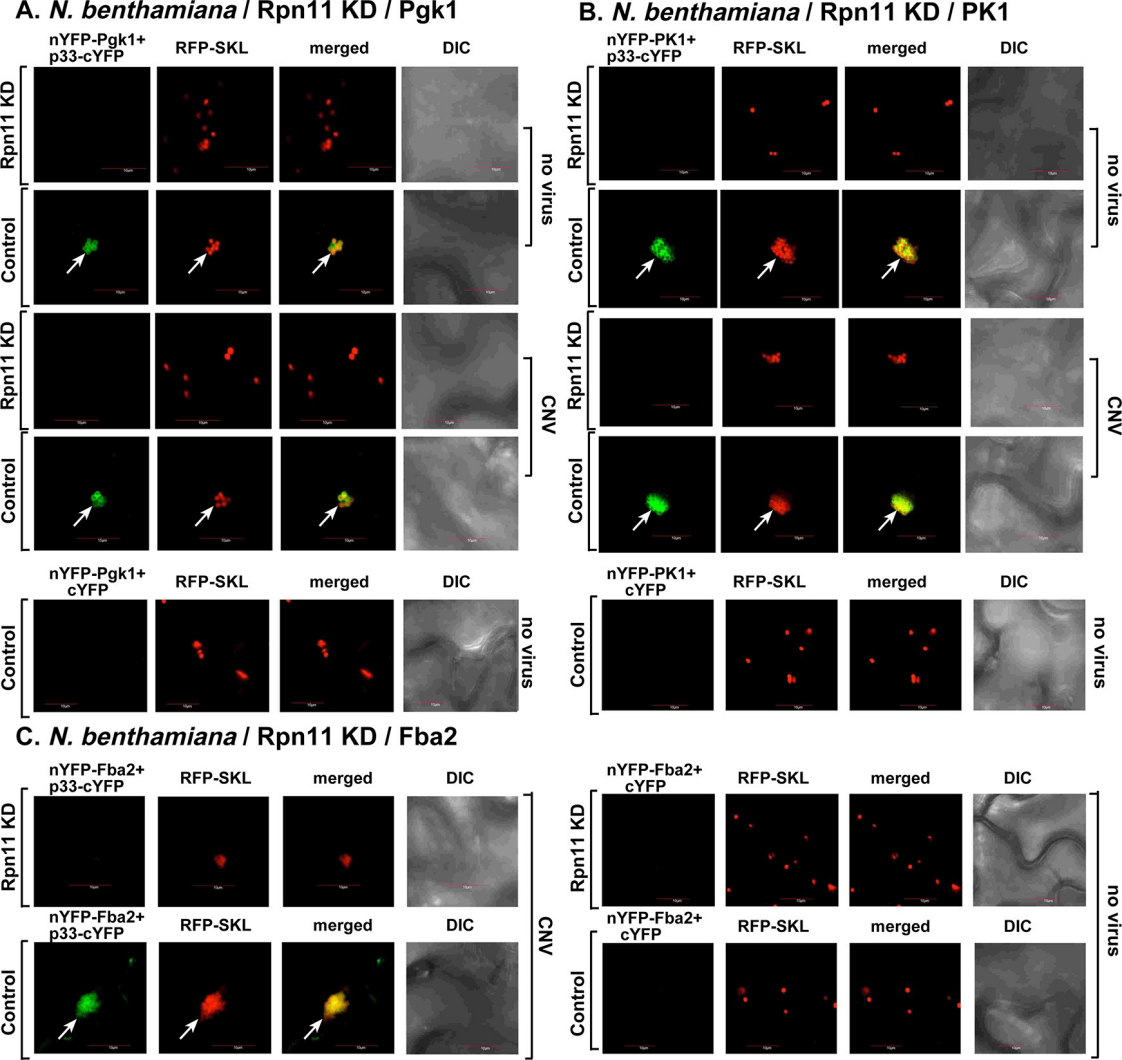
Knockdown of the proteasomal Rpn11 deubiquitinase inhibits and recruitment of glycolytic enzymes Pgk1, PK1 and Fba2 into the VROs and their interactions with p33 replication protein. (A) VIGS of Rpn11 mRNA level affects the interaction between TBSV p33 replication protein and the cellular Pgk1. *N*. *benthamiana* plants were silenced with VIGS vector targeting a region of Rpn11 for 8.5 days. Then, the leaves were co-agroinfiltrated with BiFC plasmids pGD-nYFP-Pgk1 and pGD-p33-cYFP as well as pGD-RFP-SKL to express peroxisomal matrix marker to indicate VROs. Confocal images were taken 1.5 days after agroinfiltration. The control experiments included the TRV2-nMBP VIGS plasmid. The merged images show the colocalization of the BiFC signal with the peroxisomal marker, indicating the interaction between TBSV p33 replication protein and Pgk1 within VROs. The plants were also agroinfiltrated with pGD-CNV or pGD vector (as a control). (B-C) VIGS of Rpn11 mRNA level affects the interaction between p33 and the glycolytic PK1 or Fba2 enzymes. See more details in panel A. Scale bar is 10 μm. Each experiment was repeated.

**Fig 2:**
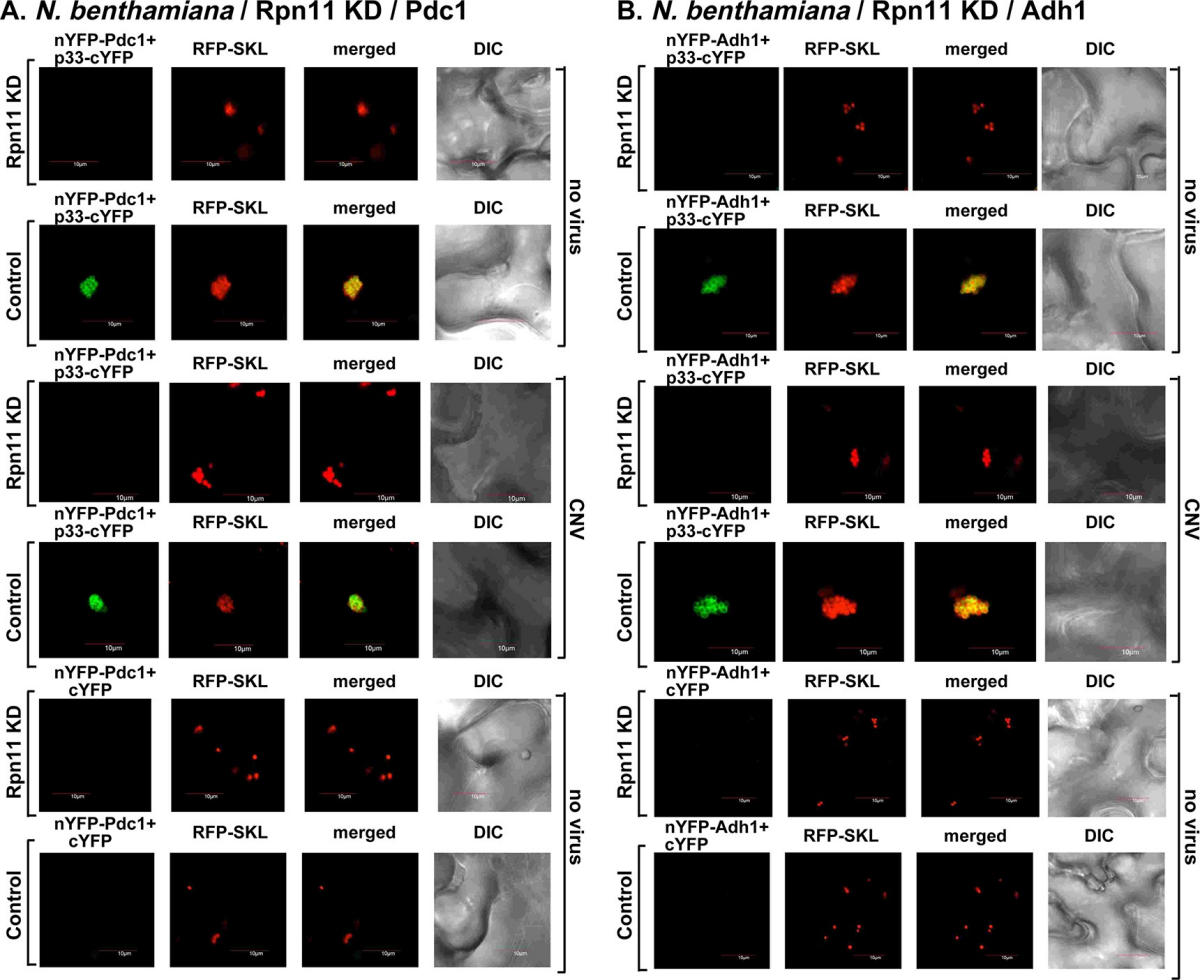
Knockdown of the proteasomal Rpn11 reduces recruitment of fermentation enzymes Pdc1 and Adh1 into the VROs. (A-B) VIGS of Rpn11 mRNA level affects the interaction between TBSV p33 replication protein and the cellular Pdc1 and Adh1. *N*. *benthamiana* plants were silenced with VIGS vector targeting a region of Rpn11 for 8 days. Then, the leaves were co-agroinfiltrated with BiFC plasmids pGD-nYFP-Pdc1 or nYFP-Adh1 and pGD-p33-cYFP as well as pGD-RFP-SKL. Confocal images were taken 1.5 days after agroinfiltration. See more details in Fig 1A. Scale bar is 10 μm. Each experiment was repeated.

We performed BiFC assays, which are suitable to determine protein-protein interactions and the subcellular location of the interactions if cellular markers are also co-expressed [58]. Based on the BiFC experiments, we did not observe interaction between p33 and Pgk1, PK1 and Fba2 glycolytic enzymes within the p33-induced VRO-like structures in *N*. *benthamiana* after VIGS treatment that knocked down Rpn11 mRNA level (Fig 1A and 1B and 1C). The BiFC results were comparable when the plants were also infected with cucumber necrosis virus (CNV, closely related to TBSV) to induce functional VROs (Fig 1). This is in contrast with the efficient p33-Pgk1, p33-PK1 and p33-Fba2 interactions within the large VROs decorated with the RFP-SKL peroxisomal marker in the control plants (Fig 1). We also observed the reduced level of peroxisome aggregation in the Rpn11 knockdown plants in comparison with the high level peroxisomal aggregation in the control plants, which is a characteristic feature of tombusvirus VROs (Fig 1) [59, 60]. Pgk1, PK1 and Fba2 proteins were expressed in Rpn11 knockdown plants (S1B Fig). In addition, we observed the lack of detectable interaction via BiFC between p33-Pdc1 and p33-Adh1 fermentation enzymes in Rpn11 knockdown plants infected with CNV or mock-infected (Fig 2A and 2B). This is in contrast with the robust interactions between p33 and the fermentation enzymes within VROs in control plants (Fig 2A and 2B). Pdc1 and Adh1 proteins were expressed in Rpn11 knockdown plants (S1B Fig). Altogether, these data indicated that reduced expression of Rpn11 led to poor recruitment of selected glycolytic and fermentation enzymes by p33 into tombusvirus VROs in *N*. *benthamiana* plants.

To provide further evidence on the role of Rpn11 as the key regulator of recruitment of pro-viral cytosolic enzymes into VROs, we retargeted a bulk fraction of Rpn11 from the cytosol into the nucleus in *N*. *benthamiana*. This was achieved through incorporating a nuclear retention signal (NRS) into Rpn8 proteasomal protein, which is a strong interactor with Rpn11 in the proteasomal lid [61–63]. Originally, Rpn8 and Rpn11 are distributed in both cytosol and the nucleus (S2 Fig). GFP-NRS-Rpn8, however, was exclusively retained in the nucleus marked by RFP-tagged histone H2B (S2A Fig). The ectopic expression of GFP-NRS-Rpn8 resulted in efficient accumulation of RFP-Rpn11 also in the nucleus (S2C Fig). Interestingly, expression of p33 replication protein resulted in partial recruitment of GFP-Rpn8 into the VROs, whereas GFP-NRS-Rpn8 was exclusively retained in the nucleus in *N*. *benthamiana* infected with CNV (S2B Fig). Based on these data, we have developed a new approach to sequester Rpn8 and co-sequester Rpn11 into the plant nucleus.

**Fig 3:**
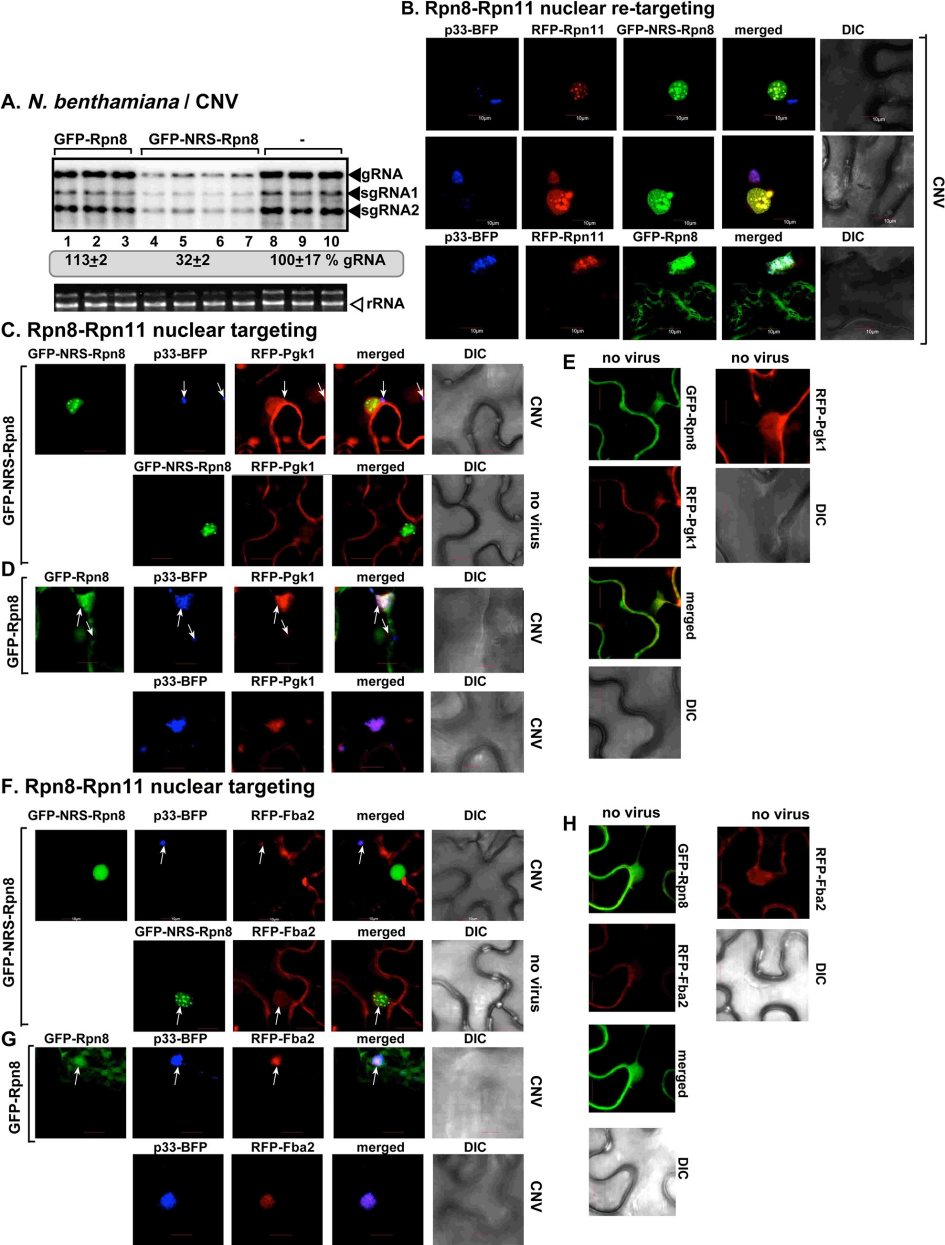
Sequestration of Rpn11 from the cytosol to the nucleus inhibits recruitment of glycolytic enzymes into the VROs. (A) Top panel: Accumulation of the CNV genomic (g)RNA in *N*. *benthamiana* plants expressing GFP-NRS-Rpn8 2.5 days post-inoculation (dpi) in the inoculated leaves was measured by northern blot analysis. Second panel: Ribosomal RNA is shown as a loading control in an ethidium-bromide stained agarose gel. Each experiment was repeated three times. (B) Sequestration of RFP-Rpn11 via expression of GFP-NRS-Rpn8 inhibits co-localization between TBSV p33-BFP replication protein and Rpn11. *N*. *benthamiana* plants were inoculated with CNV. Control experiments included plants expressing GFP-Rpn8. (C) Expression of GFP-NRS-Rpn8 inhibits co-localization between TBSV p33-BFP replication protein and RFP-Pgk1. Arrows point at small VROs marked by p33-BFP. (D) Expression of GFP-Rpn8 does not change co-localization between TBSV p33-BFP replication protein and RFP-Pgk1 in *N*. *benthamiana* plants infected with CNV. (E) Confocal images from control plants lacking tombusviral components. (F) Expression of GFP-NRS-Rpn8 inhibits co-localization between TBSV p33-BFP replication protein and RFP-Fba2. (G) Expression of GFP-Rpn8 does not change co-localization between TBSV p33-BFP replication protein and RFP-Fba2 in *N*. *benthamiana* plants infected with CNV. (H) Confocal images from control plants expressing RFP-Fba2, but lacking tombusviral components. Scale bar is 10 μm. Each experiment was repeated.

Expression of GFP-NRS-Rpn8 inhibited CNV replication by ~3-fold, likely due to co-sequestration of Rpn11 into the nucleus (Fig 3A). On the contrary, ectopic expression of GFP-Rpn8 did not affect CNV replication (Fig 3A). Confocal microscopy analysis revealed that RFP-Rpn11 was inefficiently recruited into VROs in *N*. *benthamiana* expressing GFP-NRS-Rpn8 and infected with CNV (Fig 3B). This is in contrast with the efficient recruitment of RFP-Rpn11 into VROs expressing GFP-Rpn8 and infected with CNV (Fig 3B). Next, we tested the recruitment of the glycolytic Pgk1 and Fba2 into VROs. Expression of GFP-NRS-Rpn8 remarkably inhibited the recruitment of RFP-Pgk1 (Fig 3C, top image) and RFP-Fba2 (Fig 3F, top image) into VROs in CNV-infected *N*. *benthamiana*. This is in contrast with the efficient recruitment of RFP-Pgk1 (Fig 3D, top image) and RFP-Fba2 (Fig 3G, top image) into VROs in plants expressing GFP-Rpn8 (Fig 3D). As controls, expression of GFP-Rpn8 did not change the cytosolic localization of RFP-Pgk1 or RFP-Fba2 (Fig 3E and 3H).

**Fig 4:**
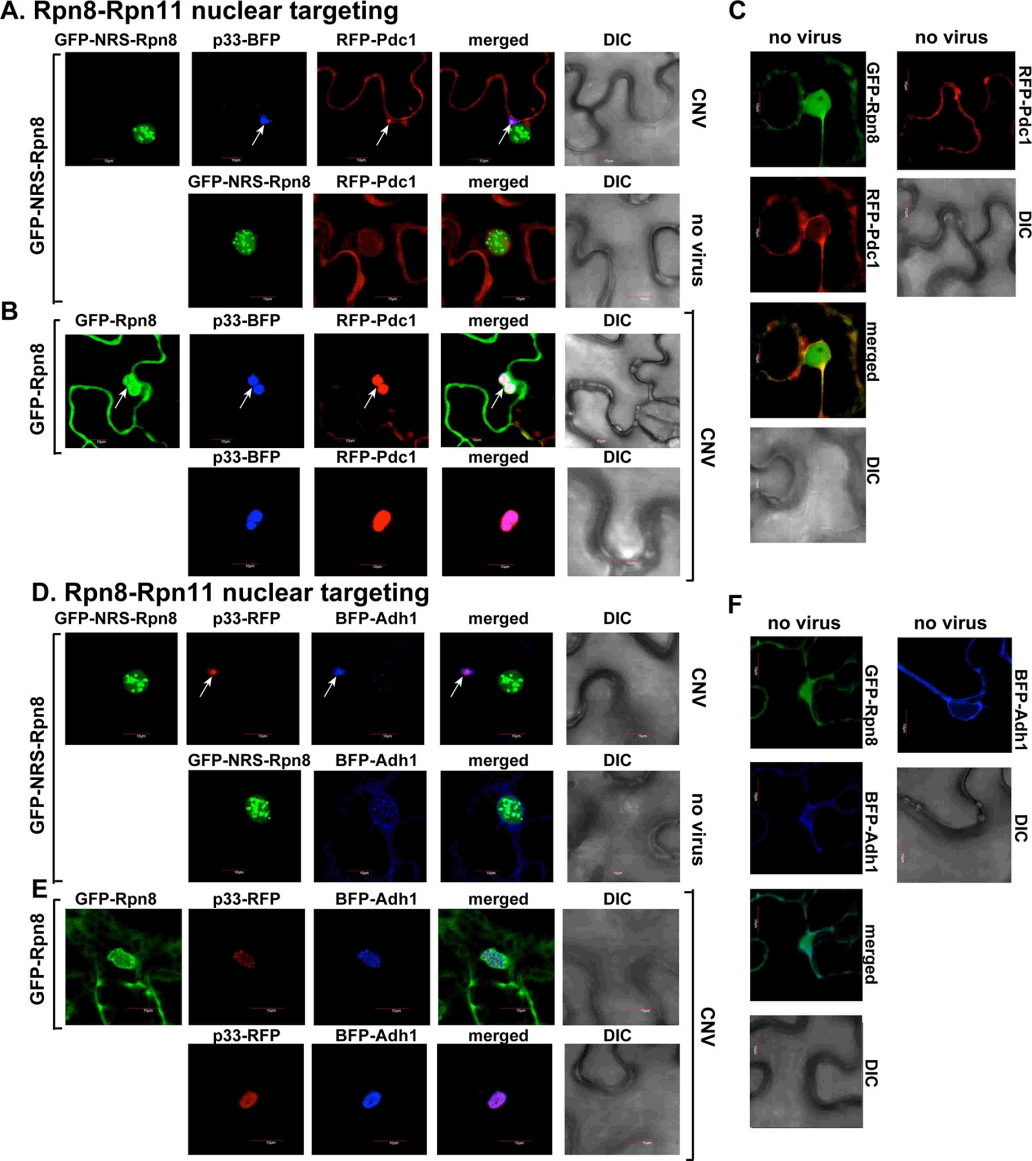
Sequestration of RFP-Rpn11 via expression of GFP-NRS-Rpn8 inhibits co-localization between TBSV p33-BFP replication protein and fermentation enzymes. *N*. *benthamiana* plants were inoculated with CNV. Control experiments included plants expressing GFP-Rpn8. See further details in Fig 3. Scale bar is 10 μm. Each experiment was repeated.

We observed similar inhibition of recruitment of the fermentation enzymes Pdc1 (Fig 4A), and Adh1 (Fig 4D) into VROs in *N*. *benthamiana* expressing GFP-NRS-Rpn8 and infected with CNV. Both these fermentation enzymes are efficiently recruited into VROs in *N*. *benthamiana* expressing GFP-Rpn8 and infected with CNV (Fig 4B and 4E). Overall, these data revealed that sequestration of Rpn11 into the nucleus via GFP-NRS-Rpn8 inhibited the recruitment of selected glycolytic and fermentation enzymes by p33 into tombusvirus VROs in *N*. *benthamiana* plants.

**Fig 5:**
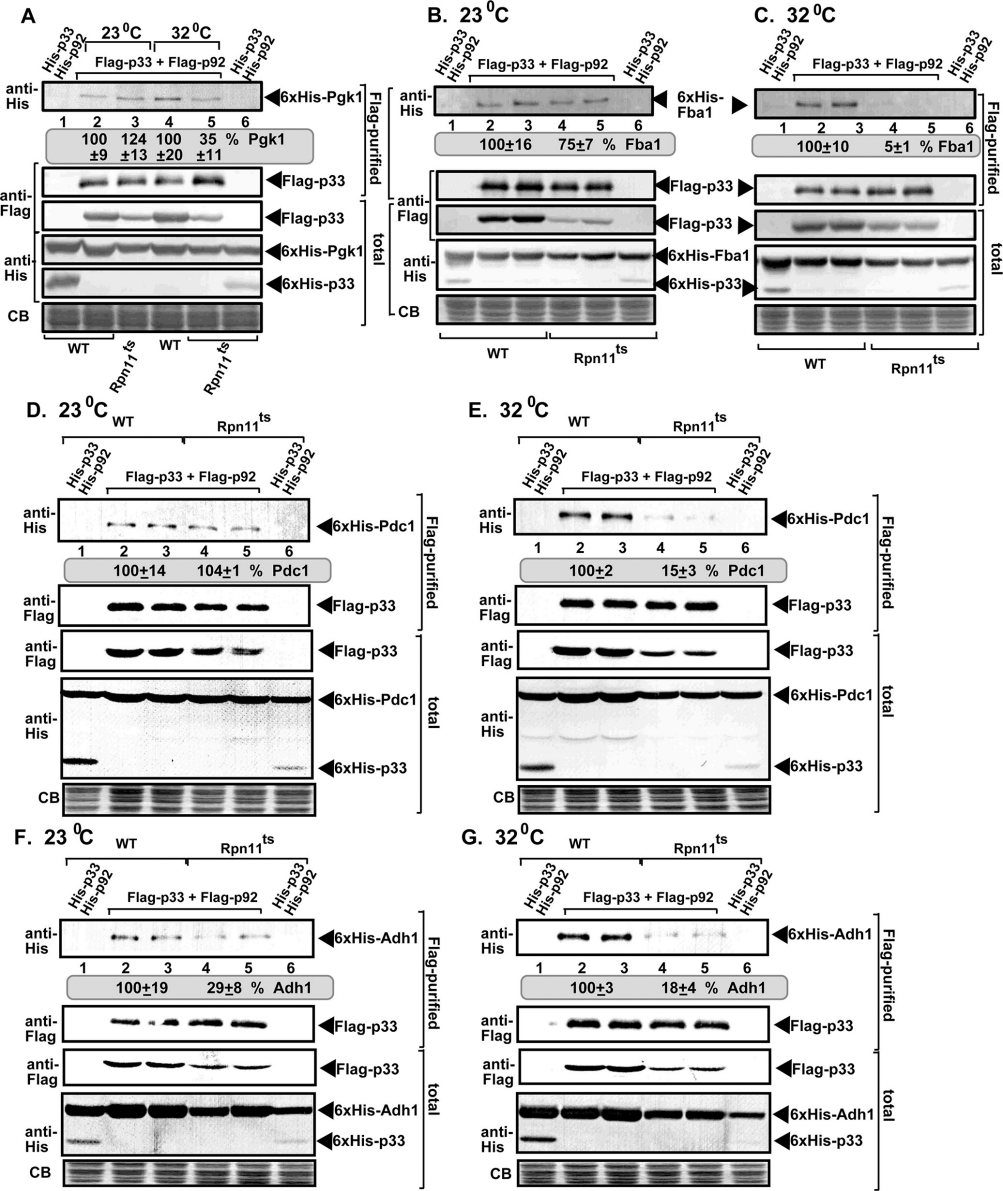
Temperature-sensitive mutation in Rpn11 reduces the co-purification of glycolytic and fermentation enzymes with the viral replicase. (A) Flag-p33 and Flag-p92 replication proteins and the TBSV repRNA were expressed in WT and *rpn11-14*^*ts*^ yeasts together with His_6_-Pgk1. First panel: Western blot analysis of co-purified His_6_-Pgk1 with TBSV replicase from detergent-solubilized membrane fraction of yeast cultured at either permissive (23°C) or semi-permissive (32°C) temperatures. Pgk1p was detected by western blot with anti-His antibody. Second panel: Western blot shows Flag-affinity purified p33 in the same samples as above with anti-Flag antibody. Third panel: Western blot analysis shows the levels of Flag-p33 in total protein extracts detected with anti-Flag antibody. Fourth panel and Fifth panel: Western blots of His_6_-Pgk1 and His_6_-p33 in total protein extracts detected with anti-His antibody. Sixth panel: Coomassie-blue stained gel SDS gel of the total protein extracts as loading controls. (B-C) Co-purification of the glycolytic Fba1 enzyme with the viral replicase. WT and *rpn11-14*^*ts*^ yeasts co-expressed Flag-p33 and Flag-p92 replication proteins and the TBSV repRNA together with His_6_-Fba1. See further details in panel A. (D-G) Co-purification of yeast His_6_-Pdc1 and His_6_-Adh1 with the viral replicase complex from WT and *rpn11-14*^*ts*^ yeasts cultured at permissive and semi-permissive temperatures. See further details in panel A. Each experiment was repeated three times and standard error is calculated.

To confirm the above findings, we utilized a temperature-sensitive (ts) Rpn11 yeast strain [43, 64]. The yeast His_6_-tagged Fba1 (a homolog of the plant Fba2, Fig 5B and 5C), His_6_-Pdc1 (Fig 5D and 5E) and His_6_-Adh1 (Fig 5F and 5G) were poorly co-purified with the Flag-tagged p33 and Flag-p92^pol^, representing the tombusvirus replicase from detergent-solubilized membrane fraction of rpn11^ts^ yeast cultured at the semi-permissive temperature (i.e., 32°C) in comparison with the WT yeast. However, the above host proteins were as efficiently co-purified with the tombusvirus replicase from rpn11^ts^ yeast cultured at the permissive temperature (i.e., 23°C) as from WT yeast (Fig 5), albeit the amount of Flag-p33 expressed was slightly lower in the rpn11^ts^ yeast. Co-purification of His_6_-Pgk1 with the tombusvirus replicase from rpn11^ts^ yeast cultured at the semi-permissive temperature was also lower than from WT yeast (Fig 5A). This was also observed with Tdh2 and Tdh3 NADH-producing glyceraldehyde-3-phosphate dehydrogenese (GAPDH, called Tdh2/3 in yeast) (S3 Fig). The enhanced co-purification of the pro-viral His_6_-RH2 DEAD-box helicase [12] with the tombusvirus replicase from rpn11^ts^ yeast cultured at the semi-permissive temperature shows that p33-based recruitment of not all cytosolic host proteins is dependent on Rpn11 (S3B Fig). Altogether, both the plant- and yeast-based data strongly support the critical role of the cytosolic Rpn11 in assisting tombusviruses during recruitment of pro-viral glycolytic and fermentation enzymes from the cytosol into VROs.

### The co-opted Rpn11 facilitates ATP production within tombusvirus VROs

**Fig 6:**
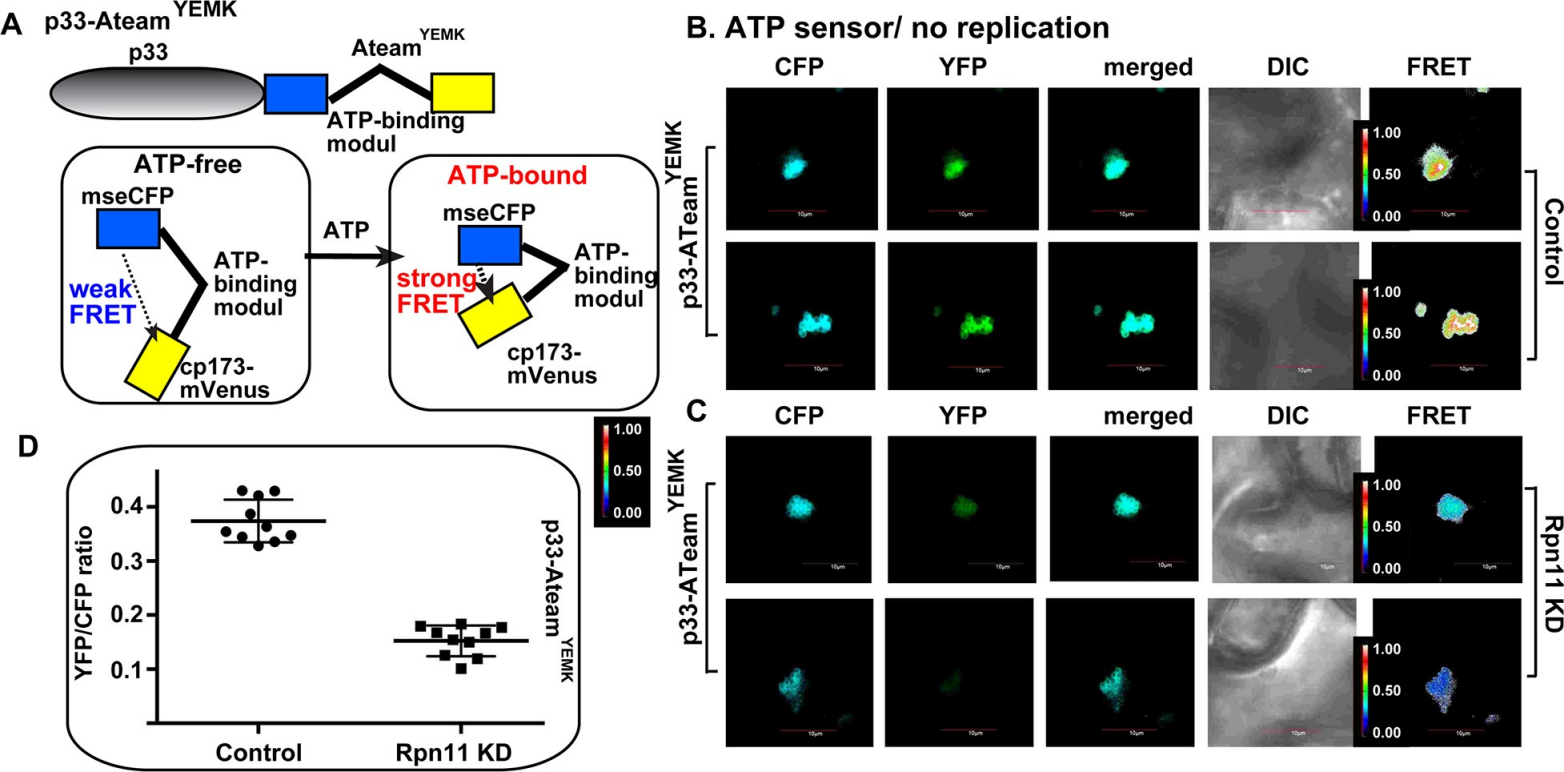
Dependence of ATP generation on Rpn11 level within tombusvirus VROs in plants. (A) A scheme of the FRET-based detection of ATP within the tombusvirus replication compartment. The enhanced ATP biosensor, ATeam^YEMK^ was fused to TBSV p33 replication protein. See further details in the main text. (B-C) Comparison of the ATP levels produced within the tombusvirus VROs in *N*. *benthamiana* treated with control or VIGS constructs (for 8 days) to knockdown Rpn11. Plants were also expressed p33-ATeam^YEMK^. The more intense FRET signals are white and red (between 0.5 to 1.0 ratio), whereas the low FRET signals (0.1 and below) are light blue and dark blue. Scale bars represent 10 μm. Each experiment was repeated three times. (D) We show the quantitative FRET values (obtained with ImageJ) for a number of samples in the graph.

The recruited glycolytic and fermentation enzymes are exploited by TBSV to produce ample amount of ATP locally within the VROs [19–21, 54]. To confirm the key role of Rpn11 in ATP production *in situ* in TBSV VROs, we used a FRET-based ATP-biosensor [65], which was previously adapted to estimate ATP levels within VROs [20, 21]. The ATP-biosensor is based on a fusion protein, linking the ATP-sensor module with p33 replication protein (called p33-ATeam) (Fig 6A). Upon binding to ATP, p33-ATeam increases FRET signal, which is measured by confocal laser microscopy. The localization of p33-ATeam to the VROs allows for the estimation of ATP level within the VROs. We found that the TBSV VROs in Rpn11 knockdown plants produced ~3 times less ATP within VROs than in the control *N*. *benthamiana* plants (Fig 6C and 6D versus 6B). These results connected the role of Rpn11 in recruitment of glycolytic and fermentation enzymes with ATP production within tombusvirus VROs in plant cells.

### The actin filaments play a key role in co-opting Rpn11 into tombusvirus replication and VRO formation

Because Rpn11 interacts with the actin network in yeast cells [48, 49], and the actin network is co-opted by tombusviruses to build the VROs [50], we decided to test the possible combined and coordinated role of Rpn11 and the subverted actin network in VRO biogenesis.

First, we applied a new approach to manipulate the actin network in plant cells infected with TBSV. This was based on two *Legionella* bacterium effectors, namely VipA and RavK, which alter the actin filaments differently. VipA is an actin nucleator, which promotes stable actin filaments [66, 67]. On the other hand RavK is a protease, which cleaves off actin monomers from the actin filaments [68]. However, the cleavage by RavK results in a nonfunctional actin monomer that cannot be reused to build new actin filaments. This process thus leads to the destruction of most of the actin filaments in cells [68].

**Fig 7:**
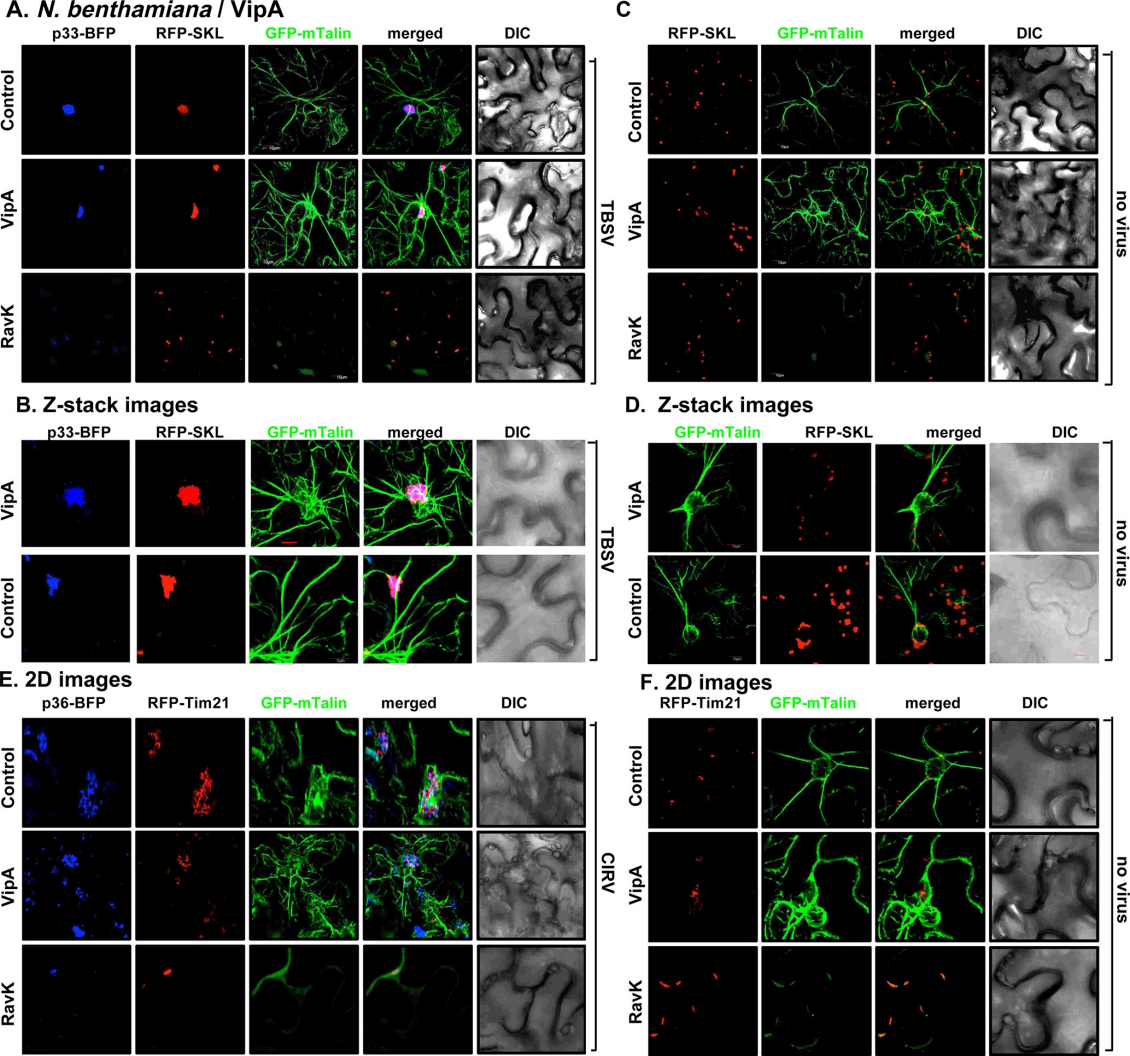
Ectopic expression of *Legionella* VipA and RavK effectors change the architecture of the actin filaments and influence the formation of VROs in *N*. *benthamiana*. (A) Transgenic *N*. *benthamiana* plants expressing GFP-mTalin were co-agroinfiltrated with pGD-p33-BFP and pGD-RFP-SKL to visualize the VROs. Confocal images show the localization of VROs in comparison with the actin filaments. *N*. *benthamiana* plants expressing VipA or RavK or neither of them as a control were inoculated with TBSV 16 h after agroinfiltration. Confocal images were taken 1.5 dpi. (B) Z-stack images of GFP-mTalin plants infected with TBSV expressing VipA. See more details in panel A. (C-D) Control confocal images show the arrangement of the actin filaments in the GFP-mTalin plants in the absence of viral components. These experiments were performed at the same time as those shown in panel A-B. (E) Transgenic GFP-mTalin *N*. *benthamiana* plants expressing CIRV p36-BFP and RFP-Tim21 mitochondrial marker to visualize VROs were analyzed by confocal imaging as described in panel A. Scale bar is 10 μm. Each experiment was repeated three times.

Transient expression of *Legionella* VipA in *N*. *benthamiana* leaves infected with TBSV resulted in the formation of the characteristic VROs decorated with p33-BFP and consisting of aggregated peroxisomes (decorated with RFP-SKL) (Fig 7A). The sizes of VROs frequently looked larger than those VROs formed in plants infected with TBSV in the absence of VipA expression (Figs 7B, Z-stack images, and S4A). The actin filaments were abundant in TBSV-infected cells and in VipA expressing cells (Figs 7A and 7B and S4A and S4B). The combination of VipA expression and TBSV infection seems to lead to the most abundant actin filaments and also the thickest ones, representing actin cables (Figs 7A and 7B and S4A and S4B).

On the contrary, transient low-level expression of *Legionella* RavK effector in *N*. *benthamiana* leaves infected with TBSV greatly inhibited VRO formation and the abundance of the actin filaments (Fig 7A). Interestingly, the p33 replication protein was still localized to the peroxisomes, which were not intensively aggregated in TBSV-infected cells, when RavK was expressed (Fig 7A). VipA and RavK expression affected the actin filament formation in the absence of viral components (Fig 7C and 7D). Based on these results, we suggest that RavK expression inhibits TBSV VRO formation via destruction of the actin filaments.

Testing the mitochondria-associated CIRV, we observed similar phenomenon, including (i) that the VipA-driven stabilization of actin filaments did not inhibit CIRV VRO formation and mitochondrial aggregation within VROs (Fig 7E), but frequently resulted in enlarged-sized CIRV VROs when VipA is co-expressed in plants (S4C and S4D Fig). The control image is shown in Fig 7E, top image panel; (ii) the RavK-based destruction of the actin filaments greatly inhibited VRO formation and the p36 replication protein-driven aggregation of mitochondria in CIRV-infected plant cells (Fig 7E). Therefore, it seems that affecting the actin filaments by the *Legionella* VipA and RavK, respectively, influenced TBSV and CIRV VRO biogenesis in *N*. *benthamiana*.

**Fig 8:**
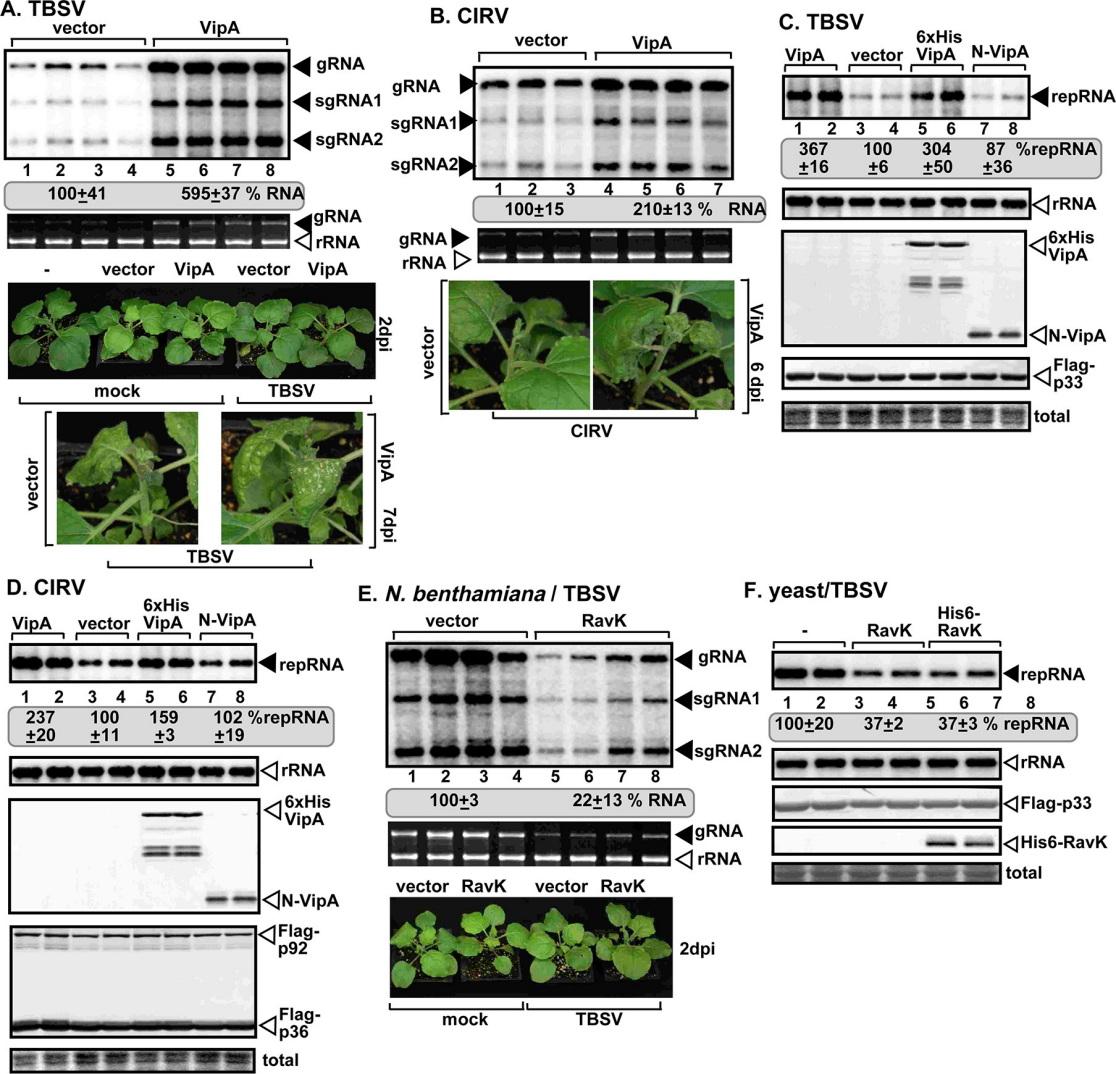
Ectopic expression of *Legionella* VipA and RavK effectors affect tombusvirus replication in plants and yeast. (A-B) Enhanced TBSV and CIRV gRNA accumulation in plant leaves expressing VipA. *N*. *benthamiana* plants were agroinfiltrated with pGD vector (control) or pGD-VipA. Agroinfiltrated plant leaves were inoculated with TBSV and CIRV 16 h after agroinfiltration. Plant samples were collected 2 dpi for samples in panel A and 3 dpi for samples in panel B, respectively. Northern blots show the increased accumulation of TBSV and CIRV gRNA in plant leaves expressing VipA. Second panel: Ethidium bromide-stained agarose gel of plant ribosomal RNA was used as loading control. Third panel: Pictures of *N*. *benthamiana* plants expressing VipA showed no phenotype and no viral symptoms when the samples were collected 2–3 dpi. Fourth panel: Pictures taken 6 (panel A) and 8 dpi (panel B) show enhanced symptoms in plants expressing VipA. (C-D) Northern blots show higher accumulation of TBSV or CIRV RNAs in yeast cells expressing VipA. Top panel: Untagged VipA, His_6_-tagged VipA, and His_6_-N-VipA mutant were expressed in yeast. Protein expression was induced with galactose for 24 h. Viral proteins Flag-p33 and Flag-p92^pol^ were expressed from plasmids from *CUP1* promoter and DI-72 (+)repRNA was expressed from *GAL1* promoter. Second panel: Yeast 18S ribosomal RNA was used as loading control. Third panel: His_6_-VipA effector and the mutants expressed in yeast were detected with anti-His antibody. Fourth panel: Flag-p33 was detected with Anti-Flag antibody. Fifth panel: Coomassie blue-stained SDS-PAGE gel shows total protein extracts. (E) Transient expression of RavK effector reduces TBSV gRNA accumulation in plants. *N*. *benthamiana* plants were agroinfiltrated with pGD vector or pGD-RavK. 16 h later, the agroinfiltrated leaves were inoculated with TBSV. Total RNA samples were analyzed at 2 dpi. There is no phenotype in the plants expressing RavK and no visible TBSV-induced symptoms. See additional details in panel A. (F) Northern blot shows reduced TBSV repRNA accumulation in yeast cells expressing RavK in comparison with control. See further details in panel D. Each experiment was repeated three times.

To test the effectiveness of the *Legionella* VipA and RavK effectors in modulation of the actin filaments on tombusvirus replication, we measured TBSV and CIRV genomic (g)RNA accumulation in *N*. *benthamiana* leaves transiently expressing the effectors. Northern blot analysis revealed 2-to-6-fold increased accumulation of tombusviruses in *N*. *benthamiana* expressing VipA effector (Fig 8A and 8B). The severity of symptoms caused by tombusviruses was also enhanced in *N*. *benthamiana* expressing VipA (Fig 8A and 8B). VipA expression in yeast also increased TBSV and CIRV repRNA accumulation by ~2-3-fold (Fig 8C and 8D). VipA mutant (i.e., N-VipA) lacking the C-terminal acting-binding domain [69] was ineffective in enhancement of TBSV and CIRV replication (Fig 8C and 8D). On the contrary, transient expression of RavK inhibited TBSV accumulation by ~5-fold in *N*. *benthamiana* leaves (Fig 8E) and ~3-fold in yeast (Fig 8F). The leaves expressing RavK and used for the studies looked normal at the time of sampling (Figs 8E and S5A and S5B).

**Fig 9:**
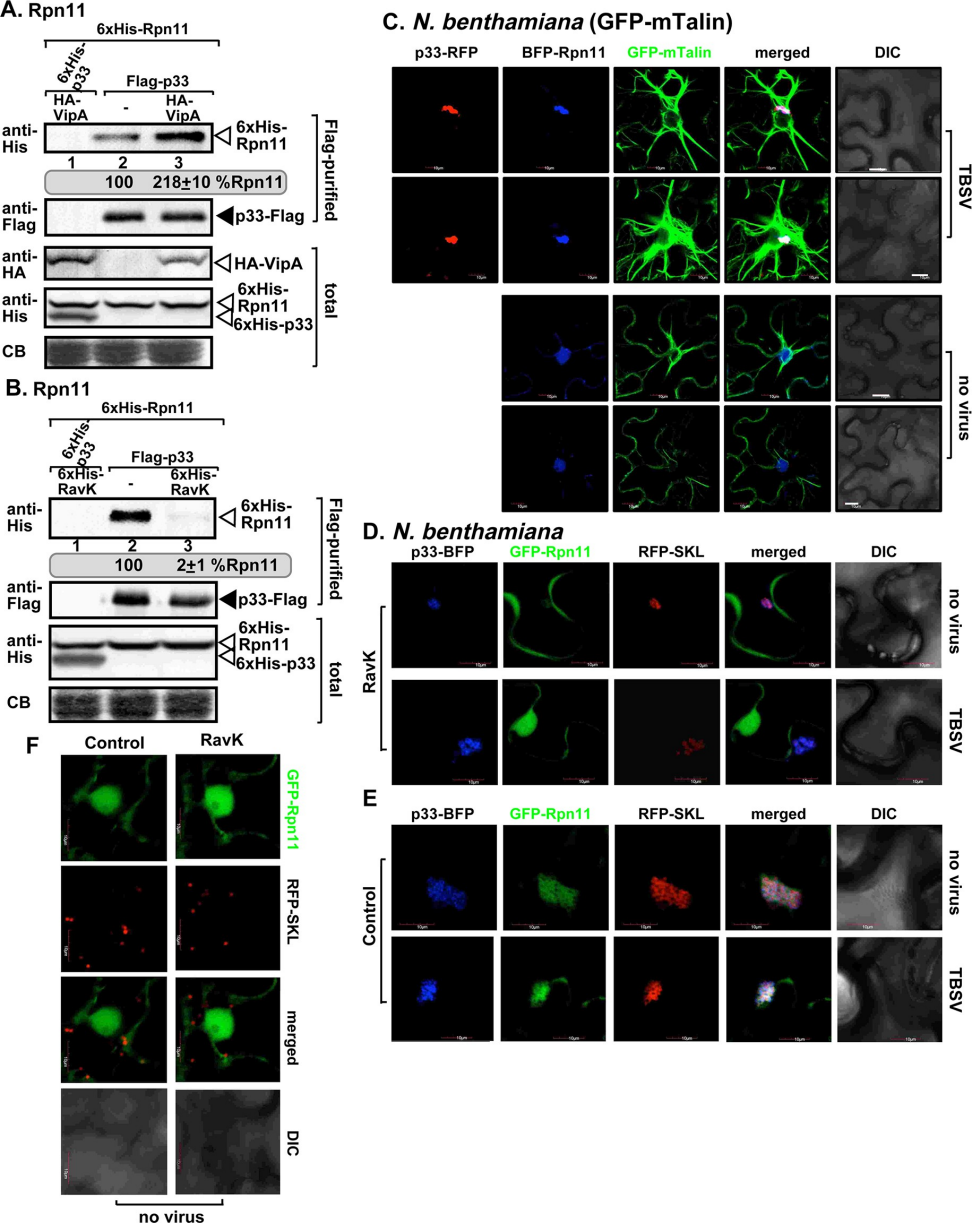
Expression of RavK effector reduces the recruitment of Rpn11 host factor into the VROs. (A) Expression of VipA effector enhances the amount of Rpn11 in viral replicase preparations purified from yeast. Co-purification of His_6_-Rpn11 with TBSV replicase (Flag-p33 and Flag-p92^pol^) from detergent-solubilized membrane fraction of yeast expressing VipA. Top panel: Western blot analysis of the co-purified His_6_-Rpn11 detected with anti-His antibody in the Flag-purified viral replicase preparations. Second panel: Western blot shows the levels of Flag-affinity purified p33 from yeast membrane fractions detected with anti-Flag antibody. Third panel: Western blot analysis shows the expression levels of 3xHA-VipA effector detected with anti-HA antibody in total protein extracts from yeast. Four panel: Western blot of His_6_-Rpn11 and His_6_-p33 in total protein extracts detected with anti-His antibody. The negative control was from yeast expressing His_6_-p33 and His_6_-p92^pol^. Fifth panel: Coomassie-blue stained gel SDS gel of the total protein extracts. (B) Expression of RavK reduces the amount of Rpn11 in viral replicase preparations purified from yeast. Top panels: Western blot analyses of the co-purified His_6_-Rpn11 and the Flag-p33 detected with anti-His and anti-Flag antibody in the purified replicase preparations. See more details in panel A. (C) BFP-Rpn11 is co-localized to the VROs and the actin filaments in transgenic *N*. *benthamina* expressing GFP-mTalin. Plants were infected with TBSV or mock-inoculated 16 h after agroinfiltration. Images were visualized at 2 dpi. (D) Confocal images of *N*. *benthamina* expressing RavK effector show poor co-localization of GFP-Rpn11 with p33-BFP and RFP-SKL in TBSV-infected or mock-inoculated leaves. (E) Confocal images of control *N*. *benthamina* show efficient co-localization of GFP-Rpn11 with p33-BFP and RFP-SKL. (F) Control confocal images with plants not expressing viral components. Scale bar is 10 μm. Each experiment was repeated three times.

Next, using the above effector protein tools, we studied if the actin network is involved in facilitating the subversion of Rpn11 into tombusvirus replication complexes. First, we Flag-affinity purified the p33 and p92^pol^ replication proteins, which are the major components of the TBSV VRCs [31], from the detergent-solubilized membrane fraction of yeast replicating TBSV repRNA and co-expressing His_6_-tagged Rpn11 and 3xHA-tagged VipA or His_6_-RavK. Western blot analysis revealed a 2-fold increase in the amount of co-purified Rpn11 in yeast co-expressing VipA (Fig 9A). Whereas, Rpn11 was barely detectable in the purified replicase preparation from yeast expressing RavK (Fig 9B). Second, we confirmed that Rpn11 co-localized with the p33 and the actin filaments in plant cells (Fig 9C). Third, we showed that expression of RavK blocked the recruitment of GFP-Rpn11 into TBSV VROs in *N*. *benthamiana* (Fig 9D), whereas GFP-Rpn11 was efficiently recruited into VROs in the control plants (Fig 9E). Expression of RavK did not affect GFP-Rpn11 distribution between the nucleus and cytosol in the absence of tombusviruses (Fig 9F). All these results establish the critical role of the actin network in subversion of Rpn11 for pro-viral functions into tombusvirus VROs.

### The actin filaments play a critical role in subversion of the cytosolic glycolytic and fermentation enzymes into tombusvirus VROs

Because recruitment of Rpn11 cytosolic protein interaction hub protein by tombusviruses depends on the actin network, as established above, and Rpn11 affects the recruitment of select group of cytosolic host factors into VROs, we assumed that modulating the activities of the actin network would have major effects on the subversion of cytosolic host factors into TBSV replication. Again, we decided to focus on the glycolytic and fermentation enzymes due their robust recruitment into VROs and the strong dependence of TBSV replication on the local generation of ATP within the VROs [54].

**Fig 10:**
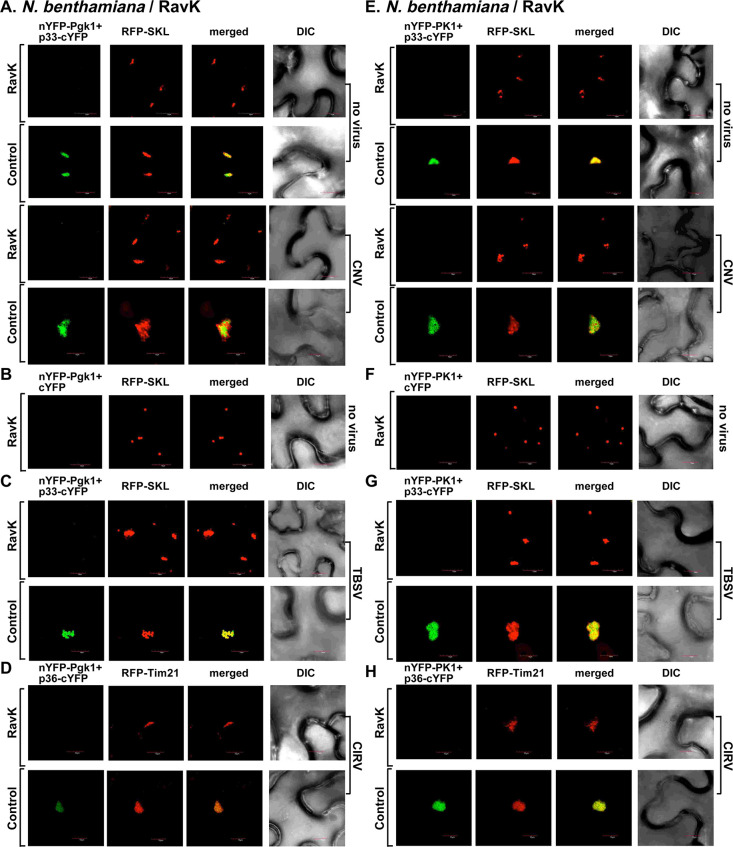
Transient expression of RavK effector inhibits recruitment of glycolytic enzymes Pgk1 and PK1 into the VROs in plants. (A) Expression of RavK effector leads to reduced interaction between tombusvirus p33 replication protein and the cellular Pgk1. *N*. *benthamiana* leaves were co-agroinfiltrated with BiFC plasmids pGD-nYFP-Pgk1 and pGD-p33-cYFP as well as pGD-RFP-SKL to express peroxisomal matrix marker to indicate VROs. Confocal images were taken 2 days after agroinfiltration. The plants were also agroinfiltrated with either pGD-CNV^20Kstop^ or pGD vector as shown. The control experiments were performed in the absence of RavK expression. The merged images show the co-localization of the BiFC signal with the peroxisomal marker, indicating the interaction between TBSV p33 replication protein and Pgk1 within VROs. (B) Negative control for the BiFC experiment shown in panel A. (C-D) Expression of RavK effector also leads to reduced interaction between TBSV p33 replication protein and the cellular Pgk1 and CIRV p36 and Pgk1. See further details in panel A. (E-F) BiFC-based experiments in *N*. *bentamiana* expressing RavK effector or controls showing the reduced interaction between tombusvirus replication protein and the cellular PK1. The experiments were performed as described in Panel A. Scale bar represents 10 μm. Each experiment was repeated three times.

**Fig 11:**
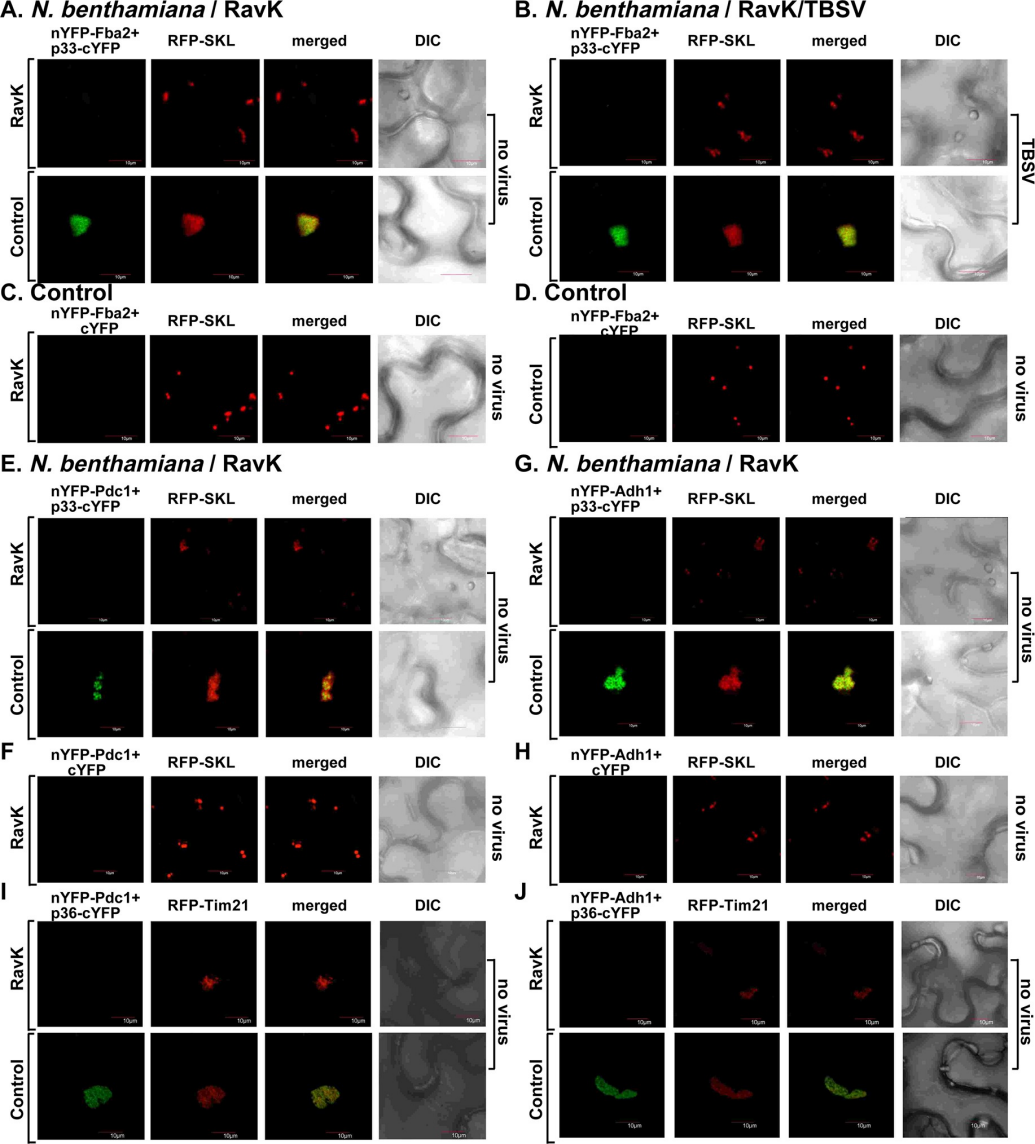
Transient expression of RavK effector inhibits recruitment of Fba2 glycolytic enzyme and Pdc1 and Adh1 fermentation enzymes into the VROs in plants. (A-B) Expression of RavK effector leads to reduced interaction between tombusvirus p33 replication protein and the cellular Fba2. *N*. *benthamiana* leaves were co-agroinfiltrated with BiFC plasmids pGD-nYFP-Fba2 and pGD-p33-cYFP as well as pGD-RFP-SKL to express peroxisomal matrix marker to indicate VROs. Confocal images were taken 2 days after agroinfiltration. The plants were infected with TBSV or mock-inoculated as shown. See further details in Fig 10, panel A. (C-D) Negative control for the BiFC experiment shown in panel A. (E-J) BiFC-based experiments in *N*. *bentamiana* expressing RavK effector showing the reduced interaction between TBSV p33 and CIRV p36 replication proteins and the cellular Pdc1 and Adh1. The experiments were performed as described in Fig 10, panel A. Scale bar represents 10 μm. Each experiment was repeated three times.

First, we transiently expressed RavK in *N*. *benthamiana* leaves and tested the interaction with p33 replication protein and the recruitment of glycolytic enzymes into VROs using BiFC. Interestingly, RavK expression inhibited the interaction between the glycolytic ATP-generating Pgk1 and PK1 (Fig 10) and Fba2 (Fig 11A and 11B) with the p33 replication protein within the VROs of CNV or TBSV. RavK expression also inhibited the recruitment of Pgk1 and PK1 into the CIRV VROs and the interaction with the p36 replication protein (Fig 10D and 10H). RavK expression led to reduction in sizes of VROs consisting of aggregated peroxisomes (marked with RFP-SKL) in case of TBSV and CNV infections as well as VROs from aggregated mitochondria in case of CIRV infection (Fig 10). Second, the transient expression of RavK inhibited the interaction with the replication proteins and subversion of Pdc1 and Adh1 fermentation enzymes into TBSV and CIRV VROs in *N*. *benthamiana* leaves (Fig 11). The Pgk1, PK, Fba2, Pdc1 and Adh1 proteins were expressed in *N*. *benthamiana* leaves also co-expressing RavK (S5C Fig).

**Fig 12:**
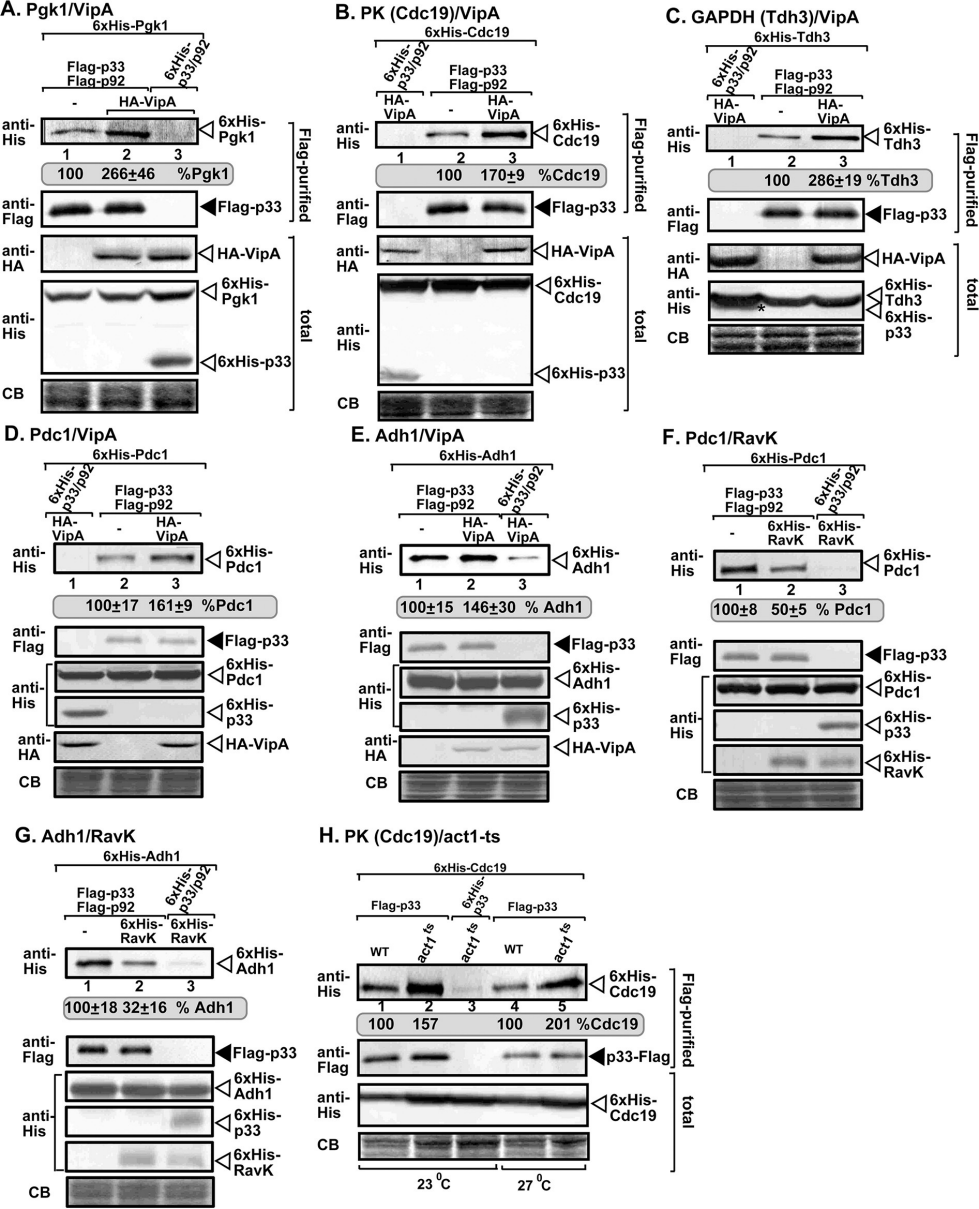
Expression of VipA and RavK effectors affect the co-purification of glycolytic and fermentation enzymes with the viral replicase in yeast. (A) Flag-p33 and Flag-p92 replication proteins were co-expressed with His_6_-Pgk1 and 3xHA-tagged VipA in WT yeast. Top panel: Western blot analysis of co-purified His_6_-Pgk1 with TBSV replicase from detergent-solubilized membrane fraction of yeast with or without 3xHA-VipA expression. The co-purified His_6_-Pgk1p was detected with anti-His antibody. Second panel: Western blot shows Flag-affinity purified p33 in the same samples as above with anti-Flag antibody. Third panel: Western blot analysis shows the levels of 3xHA-VipA in total protein extracts detected with anti-HA antibody. Fourth panel: Western blots of His_6_-Pgk1 and His_6_-p33 in total protein extracts detected with anti-His antibody. Sixth panel: Coomassie-blue stained gel SDS gel of the total protein extracts as loading controls. (B-E) Co-purification of the glycolytic His_6_-Cdc19 (PK), His_6_-Tdh3 (GAPDH) and the His_6_-Pdc1 and His_6_-Adh1 fermentation enzymes with the viral replicase from yeast expressing 3xHA-VipA. See further details in panel A. (F-G). Co-purification of His_6_-Pdc1 and His_6_-Adh1 fermentation enzymes with the viral replicase from yeast expressing His_6_-RavK. See further details in panel A. (H) Flag-p33 and Flag-p92 replication proteins were expressed in WT and *act1*^*ts*^ yeasts together with His_6_-Cdc19 (PK) glycolytic enzyme. First panel: Western blot analysis of co-purified His_6_-Cdc19 with TBSV replicase from detergent-solubilized membrane fraction of yeast cultured at either permissive (23°C) or semi-permissive (27°C) temperatures. Co-purified His_6_-Cdc19 was detected with anti-His antibody. Second panel: Western blot shows Flag-affinity purified p33 in the same samples as above with anti-Flag antibody. Third panel: Western blots of His_6_-Cdc19 in total protein extracts detected with anti-His antibody. Fourth panel: Coomassie-blue stained gel SDS gel of the total protein extracts as loading controls. Each experiment was repeated three times and standard error is calculated.

Third, we used yeast to purify the tombusvirus replicase from membrane fraction of yeast expressing the VipA effector. Western blot analysis of the co-purified host proteins revealed ~2-3-fold increased levels of the glycolytic Pgk1, Cdc19 (PK), Tdh3 (GAPDH), and Pdc1 and Adh1 fermentation enzymes in the purified tombusvirus replicase preparation when yeast expressed VipA (Fig 12). On the contrary, low-level expression of RavK effector in yeast led to ~2-fold reduction of Pdc1 and Adh1 levels in the purified tombusvirus replicase preparations (Fig 12F and 12G). We confirmed the increased recruitment of Cdc19 (PK) into TBSV replicase using a yeast actin mutant (act1^ts^), which results in stabilized actin filaments (Fig 12H) [50, 70]. All these data demonstrated the key role of the actin filaments in recruitment of glycolytic and fermentation enzymes by tombusviruses.

### The actin filaments affect ATP production within tombusvirus VROs

**Fig 13:**
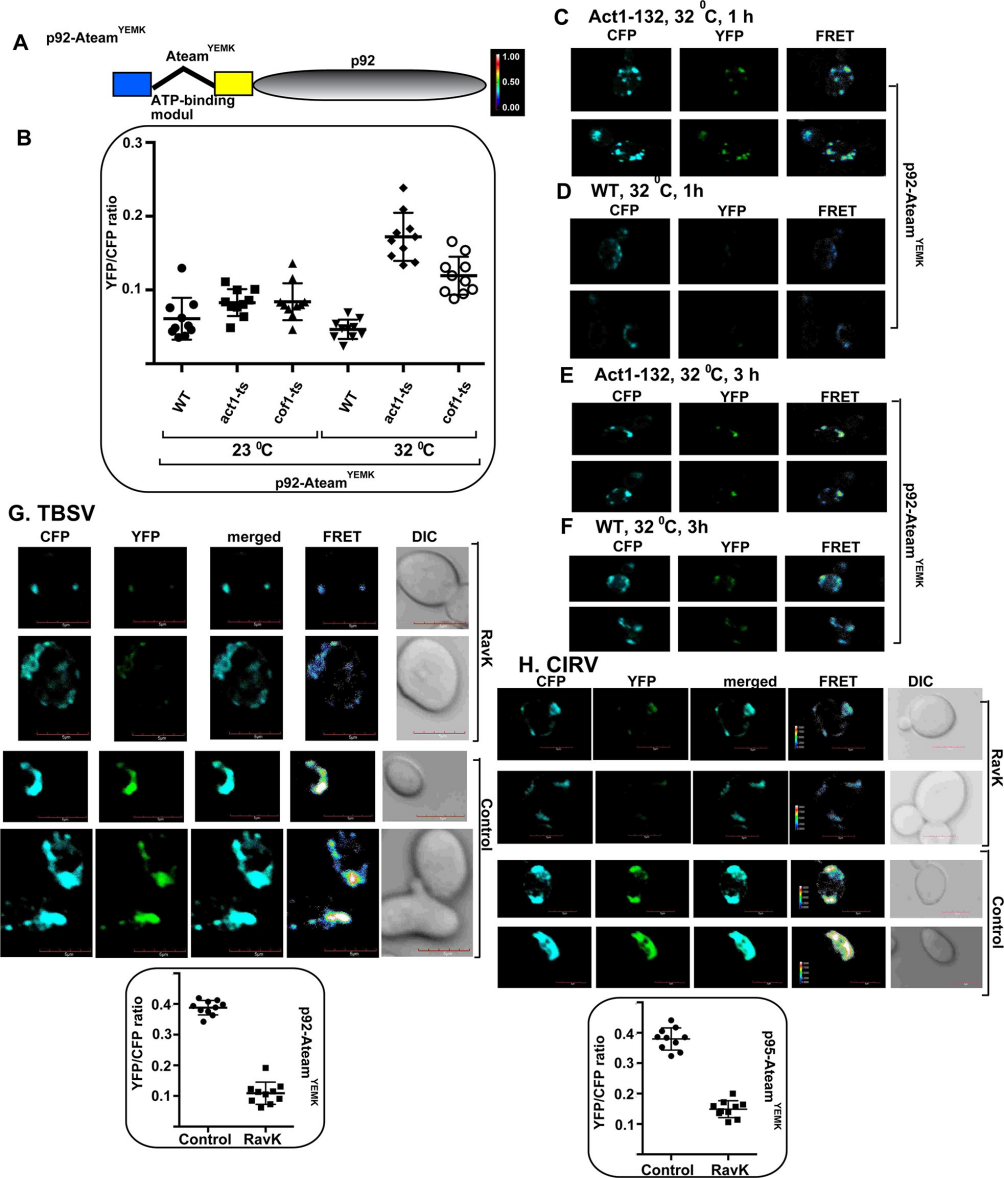
Dependence of ATP generation within tombusvirus VROs on the actin network in yeast. (A) A scheme of the FRET-based detection of ATP within the tombusvirus replication compartment. The enhanced ATP biosensor, ATeam^YEMK^ was fused to TBSV p92 replication protein. See further details in the main text. (B) Graph presentation of relative ATP levels produced within the tombusvirus VROs in WT, act1^ts^ and cof1^ts^ yeasts at the permissive and semi-permissive (32°C) temperatures. The quantitative FRET values of multiple cells were obtained with imageJ. (C-F) Comparison of ATP levels within VROs in act1^ts^ and WT yeasts after 1 or 3 h at the semi-permissive temperature. High FRET signals are red and white (between 0.5 to 1.0 ratio) and low FRET signals (between 0.1–0.5) are dark blue and light blue. (G) Comparison of relative ATP levels in TBSV VROs in yeast cells expressing RavK effector versus control yeast not expressing RavK. The graphic shows the quantitative FRET values for independent samples. Further details in panel B. (H) Comparison of relative ATP levels in CIRV VROs in yeast cells expressing RavK effector. The graphic shows the quantitative FRET values for several samples. Further details in panel B. Scale bar represents 5 μm.

To confirm that the subverted actin filaments indeed important to deliver glycolytic and fermentation enzymes to produce ATP locally within the VROs, we again used the above a FRET-based ATP-biosensor approach [19–21, 54]. The ATP-biosensor module was fused with p92^pol^ (called ATeam-p92^pol^) (Fig 13A). We found previously [20, 21] that the ATeam-tagged p92^pol^ is a fully functional RdRp. ATeam-p92^pol^ localizes to the VROs allowing the estimation of ATP level within the VROs. We found that the TBSV VROs in act1^ts^ mutant yeast produced ~4 times more ATP than in WT yeast at the semi-permissive temperature (Figs 13B and S6). In addition, a cofilin mutant yeast (cof1^ts^), which is partially deficient in actin filament depolymerization at semi-permissive temperature, also supported ~3-fold increased ATP production within the TBSV VROs (Figs 13B and S6). Time-point experiment also showed the faster ATP production within TBSV VROs in act1^ts^ yeast at the semi-permissive temperature than in WT yeast (Fig 13). On the contrary, expression of the RavK effector in WT yeast inhibited ATP production within the TBSV VROs (Fig 13G) and the CIRV VROs (Fig 13H) by ~3-to-4-fold.

**Fig 14:**
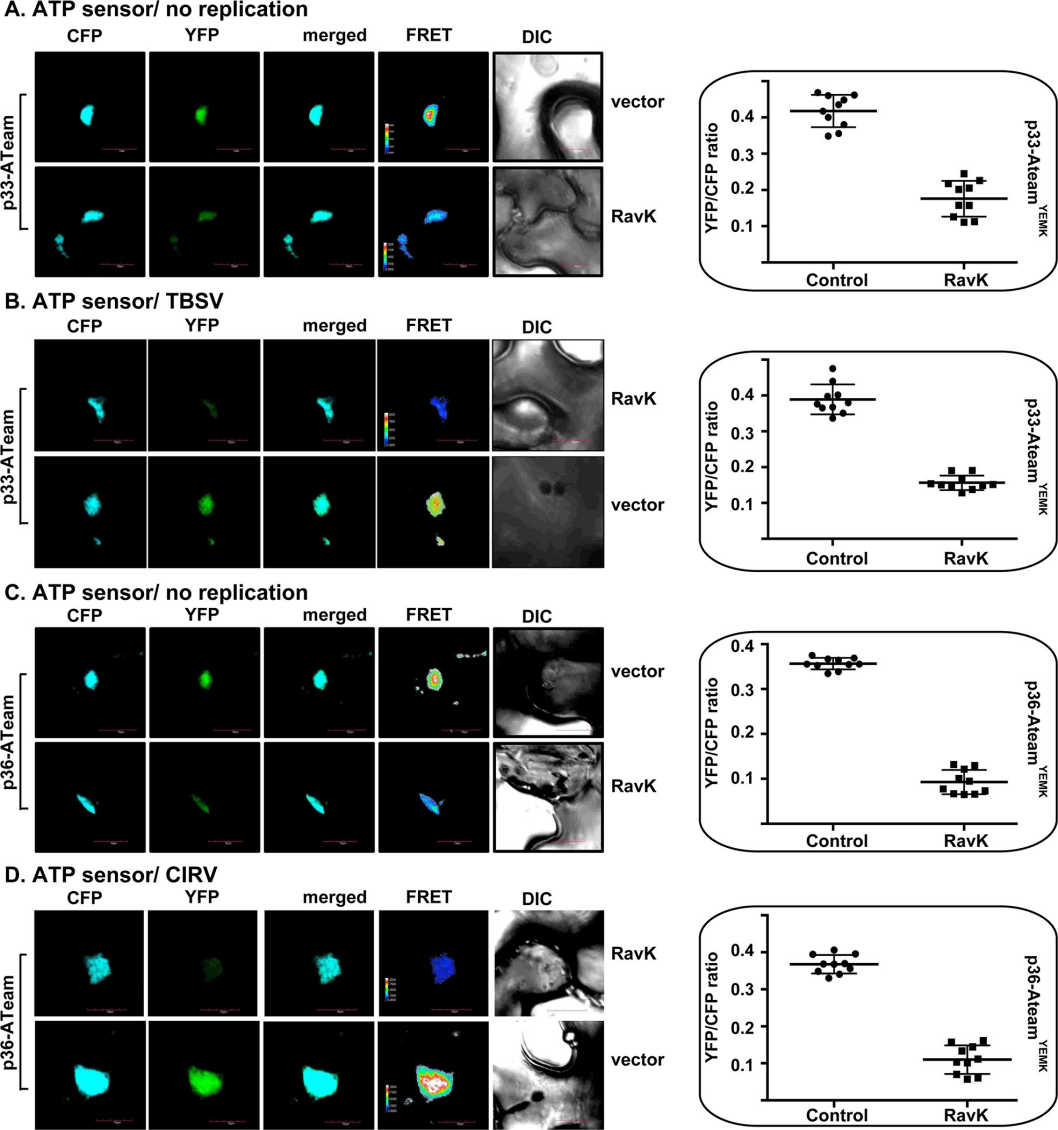
Destruction of the actin network leads to reduced ATP generation within tombusvirus VROs in plants. (A-B) ATeam^YEMK^ was fused to TBSV p33 replication protein and expressed via agroinfiltration (see Fig 6A). Comparison of relative ATP levels produced within the tombusvirus VROs in *N*. *benthamiana* expressing the *Legionella* RavK effector or the empty vector and p33-ATeam^YEMK^. The plants were infected with TBSV or mock-inoculated 16 h after agroinfiltration. FRET analysis was performed 1.5 dpi. The more intense FRET signals are white and red (between 0.5 to 1.0 ratio), whereas the low FRET signals (0.1 and below) are light blue and dark blue. We show the quantitative FRET values for a number of samples in the graph on the right. C-D). Comparable experiments in *N*. *benthamiana* expressing the *Legionella* RavK effector, but using the CIRV p36-ATeam^YEMK^ for ATP measurement within CIRV VROs. FRET analysis was performed 2 dpi. See details in panel B-C. Scale bars represent 10 μm. Each experiment was repeated three times.

The role of the actin filaments in ATP production within tombusvirus VROs is also tested in *N*. *benthamiana* plants. Expression of RavK reduced ATP production within TBSV and CIRV VROs by ~3-to-4-fold in *N*. *benthamiana* (Fig 14). Altogether, these results confirmed the essential role of the actin filaments contribution to *in situ* ATP production within tombusvirus VROs in plant cells.

## Discussion

Biogenesis of VROs is a major activity within infected cells. VRO formation requires subversion of several membranous compartments and vesicles as well as numerous co-opted cytosolic host proteins [2, 3, 5–8, 71–74]. Indeed, tombusviruses co-opt cellular membranous carriers, such as retromer-based tubular carriers, COPII vesicles and their selected cargoes via p33-based targeting of cellular membrane proteins, such as Rab1 and Rab5 small GTPases or the retromer complex and delivering them to the VROs for various functions and membrane modifications and membrane proliferation [51, 52, 75–77]. Another major function of p33 is the selection of the TBSV (+)RNA for replication and recruitment into VROs [32, 78]. However, the efficient subversion of several dozens of different cytosolic host proteins by TBSV into the VROs by a single viral protein, p33 [79, 80], raises the question: how can p33 perform so many recruitment tasks? This is in addition to performing major replication function by p33 within VROs as a structural component as well as RNA chaperone function, and the role in subversion of various membranes and membrane-bound host factors into the VROs [8, 11, 34]. One-by-one and rapid recruitment of every single cytosolic protein by the p33 replication protein would likely require a vast number of p33 molecules. Therefore, we wanted to explore if cellular cytosolic proteins might be recruited by p33 targeting of a protein interaction hub, namely the proteasomal Rpn11. Based on the presented data, we propose that Rpn11 acts as a cellular “cytosolic protein interaction hub”, which is targeted by TBSV via p33-based direct binding to subvert numerous cytosolic proteins associated with Rpn11 into VROs. In addition to the previously characterized role of Rpn11 to facilitate the recruitment of DDX3-like Ded1/RH20 DEAD-box RNA helicase into VROs [42], the current work expands the list of Rpn11-dependent co-opted host factors to four glycolytic [Pgk1, PK1 (Cdc19 in yeast), GAPDH (Tdh2/3) and Fba2 (Fba1)] and two fermentation enzymes (Pdc1 and Adh1). Using co-localization, BiFC and co-purification with the tombusvirus replicase complexes, we show the subversion of these cytosolic metabolic enzymes greatly depends on the cellular Rpn11 level or subcellular distribution or location of Rpn11, in addition to p33 replication protein. Knocking down Rpn11 level via VIGS, or sequestering of Rpn11 away from the cytosol into the nucleus via a modified retargeted Rpn8 cellular interactor protein in plants, or using a temperature-sensitive Rpn11 mutant in yeast, all provided evidence on the key role of Rpn11 in subversion of the metabolic enzymes into VROs. Rpn11 physically interacts with the above metabolic enzymes in yeast in the absence of tombusviral components [53, 81], possibly within proteasome storage granules, which form with the help of Rpn11 and predictably contain many Rpn11-interacting cytosolic proteins [45]. The glycolytic enzymes are also known to form “glycolytic or G” bodies [82, 83], which might also contribute to their efficient recruitment by TBSV into VROs. It is important to note that Rpn11 is among the most involved protein interaction hubs with documented physical interaction with ~1,000 yeast proteins (~16% of the entire yeast proteome), many of them are pro-viral host factors [8, 40, 48, 49]. We predict that the most abundant host proteins, which interact with Rpn11, will have the best chances to be recruited by p33 into VROs. The productions of many host proteins, including glycolytic and fermentation enzymes, are greatly induced by TBSV infection [19–21, 55]. The increased amounts of the induced host proteins would likely favor their associations with Rpn11. Thus, they could have the more favorable circumstances for recruitment into VROs via assistance from Rpn11 than the less abundant host proteins. Altogether, we predict that not only glycolytic and fermentation enzymes, but several more abundant cytosolic pro-viral factors might be co-opted by p33 with the help of the Rpn11 cytosolic protein interaction hub protein. The pro-viral function of Rpn11 is likely independent from its proteasomal function. Indeed, blocking proteasomal activities with MG132 inhibitor led to increased p33 replication protein level in yeast under some conditions [84]. The putative role of the proteasome system in tombusvirus replication will require future studies.

The tested metabolic enzymes are components of the aerobic glycolysis pathway, which regulates the balance between fast ATP production and biosynthesis of new biomass, including ribonucleotides, lipids and several amino acids. Tombusviruses hijack these enzymes into VROs to support local and efficient production of ATP within VROs [19–21, 54]. Their recruitments into VROs, however, apparently are affected by Rpn11.

Another major finding of this work is the involvement of the actin filaments in facilitating the TBSV p33-driven recruitment of Rpn11 cytosolic hub protein with the associated cytosolic proteins. Stabilization of the actin filaments by expression of the *Legionella* VipA effector in yeast and plant, or via mutation of *ACT1* in yeast resulted in more efficient and rapid recruitment of Rpn11 and the selected glycolytic and fermentation enzymes into VROs. On the contrary, destruction of the actin filaments via expression of the *Legionella* RavK effector led to poor recruitment of Rpn11 and glycolytic and fermentation enzymes into VROs. Ultimately, the subverted actin filaments, stabilized by TBSV p33 replication protein or VipA effector in collaboration with Rpn11 were needed for the efficient and local production of ATP by the co-opted glycolytic and fermentation enzymes within VROs representing the sites of TBSV replication in yeast and plant. Interestingly, the mitochondria associated CIRV utilizes a comparable mechanism of replication protein-driven targeting of the cytosolic Rpn11 and the actin network to deliver the glycolytic and fermentation enzymes into the VROs.

Altogether, the proposed recruitment concept for cytosolic proteins by TBSV somewhat resembles to the previously established mechanism of p33-driven subversion of targeted subcellular membranes or membrane subdomains by TBSV. For example, the small replication protein targets two membrane-associated cellular hubs, which include (i) the syntaxin18-like Ufe1 SNARE protein and Sac1 PI4P phosphatase-positive subdomains within the ER membrane, and (ii) the Rab5-positive early endosomes [51, 85–87]. The above TBSV-targeted cellular hub proteins are key parts of highly-active centers for various membrane trafficking in cells.

In summary, we present results that support a novel viral recruitment strategy for cytosolic host factors based on TBSV and CIRV. These viruses target via the small viral replication protein the cytosolic Rpn11 protein interaction hub protein and the co-opted and stabilized actin filaments. The combined and coordinated subversion of Rpn11 and the actin network allows tombusviruses to gain access to abundant cytosolic proteins, such as the glycolytic and fermentation enzymes, which are then efficiently delivered to perform pro-viral functions into the VROs, which represent the site of viral replication. Accordingly, knockdown of Rpn11 or destruction of actin filaments diminishes VRO biogenesis and tombusvirus replication in yeast and plant cells. Other (+)RNA viruses with small number of genes might also exploit similar strategies to maximize the recruitment of host factors with the involvement of limited number of viral proteins into VROs [88].

## Materials and methods

Additional Materials and Methods are presented in the supplementary information (S1 Text).

### Yeast strains

Parental yeast strain BY4741 (MATa his3Δ1 leu2Δ0 met15Δ0 ura3Δ0), was purchased from Open Biosystems. SC1 (MATa his3Δ1 leu2Δ trp1Δ289 uraΔ52) yeast strain was purchased from Invitrogen. BY4747-ADH-His92 yeast strain expressing p92 replication protein from the chromosome was made earlier [89].

### Plant and yeast expression plasmids

Plasmids used in this study are described in S1 Text.

### Tombusvirus replication assays in yeast

To test the effect of the *Legionella* VipA effector on tombusvirus replication, BY4741 yeast was co-transformed with pGBK-CUP-Flagp33/Gal-DI72, pGAD-Cup-Flagp92 or pGBK-CUP-Flagp36/Gal-DI72, pGAD-Cup-Flagp95 and one of the following plasmids: pYC-NT vector, pYC-NT-VipA [75], pAG416GAL-ccdB-VipA and pYC-N-VipA. Transformed yeasts were pre-grown in SC-ULH^−^ media supplemented with 2% glucose and BCS (from VWR) at 29°C for 16 h. Yeast cultures were washed and grown in SC-ULH^−^ supplemented with 2% galactose at 23°C for 10 h. TBSV repRNA replication was induced by adding 50 μM of CuSO_4_ for 24 h at 23°C. To test the effect of *Legionella* RavK effector on tombusvirus replication, BY4741 yeast was co-transformed with pGBK-CUP-Flagp33/Gal-DI72, pGAD-Cup-Flagp92 and one of the following plasmids: pYC-NT vector, pYC-NT-RavK, pAG416GAL-ccdB-RavK [75]. Yeast cultures were grown as above. RNA samples were analyzed by northern blot using the ^32^P-labeled DI72 RI/IV as a radioactive probe [90]. Total protein was extracted as described before [91] and protein samples were analyzed by western blot using anti-FLAG antibody to detect the viral replication proteins and anti-His antibody to detect His_6_-VipA and His_6_-RavK proteins.

### Replicase copurification assay using yeast

The tombusvirus replicase preparations were made as described before with modifications [92]. Briefly, BY4747-ADH-His92 yeast [89] was co-transformed with HpGBK-CUP1-Flagp33/Gal:DI72 or HpGBK-CUP1-Hisp33/Gal-DI72 and pRS315-Gal1-HAVipA (or pRS315-Gal1 empty vector) and one of the following plasmids: UpRS316-Tef-Pgk1, UpYES-Cdc19, UpYES-Pdc1, pYES-Adh1, UpYC-Rpn11. Sc1 yeast strain was co-transformed with HpGBK-CUP1-Flagp33/Gal:DI72, LpGAD-Trp-CUP1-Flagp92 (tryptophan selection), UpCM189-Thd3 and pRS315-Gal1-HAVipA (or pRS315-Gal1 empty vector) or HpGBK-CUP1-Hisp33/Gal-DI72, pGAD-Trp-CUP1-Hisp92 and UpCM189-Thd3 and pRS315-Gall-HAVipA.

Transformed BY4747-ADH-His92 yeast cultures were plated in SC-ULH^−^ media, whereas transformed Sc1 yeast cultures were plated in SC-ULHT^−^ media. Single colonies were streaked and grown in 20 ml of SC-ULH^−^ (or SC-ULHT^−^ in the case of Sc1 yeast) supplemented with 2% glucose and 100 μM BCS at 23°C for 16 h. Yeast cultures were washed with sterile water and grown in 40 ml of SC-ULH^−^ (or SC-ULHT^−^) supplemented with 2% galactose and 50 μM of CuSO_4_ at 23°C for 24 h. Yeast cultures were harvested and incubated in 35 ml of Phosphate Buffer Saline (PBS) containing 1% formaldehyde for 1 h on ice [93]. Formaldehyde was quenched with 0.1 M of glycine and incubated for 5 min on ice. Yeast pellets were collected and washed with PBS buffer. Flag-p33 replication proteins were purified from detergent-solubilized membrane fraction using anti-FLAG M2 agarose as described before [92]. Briefly, 0.2 g of yeast pellet was re-suspended in 200 μl of High Salt TG Buffer (50 mM Tris-HCl, pH 7.5, 10% glycerol, 15 mM MgCl_2_, 10 mM KCl) with 0.1% of yeast protease inhibitor. Yeast cells were broken and centrifuged to discard the supernatant. Yeast pellet was solubilized in High Salt TG buffer with 2% Triton X100 and 0.1% of yeast protease inhibitor. The tubes were rotated for 8 h in the cold room. Then, the tubes were centrifuged at 35,000 g for 20 min and the supernatant was transferred to an equilibrated Bio-Rad Bio-Spin chromatography column with 20 μl of FLAG M2 resin (Sigma). The column was rotated for 16 h at 4°C. The column was washed with High Salt TG Buffer and the Flag-p33 protein preparation was recovered from the column with 30 μl of SDS loading buffer. β-Mercaptoethanol was added to the eluted sample and boiled for 40 minutes to reverse the crosslink. Purified Flag-p33 preparations were analyzed by western blot and Flag-p33 protein was detected with anti-FLAG antibody. The co-purified proteins were detected with anti-His antibody. Detection with NBIP-BCIP has been described previously [91]. Protein amounts were quantified with ImageQuant software. The quantifications were analyzed in excel and standard error was calculated.

Co-purification studies were conducted from BY4747-ADH-92 yeast co-transformed with HpGBK-CUP1-Flagp33/Gal-DI72, or HpGBK-CUP1-Hisp33/Gal-DI72 as a control with pRS315-Gal-HisRavK and plasmids UpYES-Pdc1 or UpYES-Adh or UpYC-Rpn11 as above.

The haploid BY4741 and *rpn11-14*^*ts*^ yeast strains were transformed with plasmids HpGBK-CUP1-Flagp33/Gal:DI72, LpGAD-CUP1-Flagp92 and one of the following plasmids UpYES-Pdc1, pYES-Adh1 or pYC2-Fba1. Transformed yeasts were pre-grown in SC-ULH^−^ media supplemented with 2% glucose and 100 μM BCS for 16 h at 23°C (permissive temperature). Yeast cultures were transferred to SC-ULH^−^ media supplemented with 2% galactose for 24 h at 23°C or 32°C (semi-permissive temperature). Then, 50 μM of CuSO_4_ was added to the yeast cultures for 6 h. Proteins were crosslinked and Flag-p33 protein preparations were obtained from detergent-solubilized membrane fractions using anti-FLAG M2 agarose as mentioned above.

### Expression of *Legionella* effectors in *N*. *benthamiana*

*N*. *benthamiana* plants were co-agroinfiltrated with pEarleyGate100-VipA (OD_600_ 0.6) or pGD-FlagVipA (OD_600_ 0.6), together with p19 (OD_600_ 0.2) and CNV (OD_600_ 0.2). Plant samples were harvested 2.5 days post-agroinfiltration [93]. To test TBSV and CIRV replication, *N*. *benthamiana* leaves were co-agroinfiltrated with pGD-FlagVipA (OD_600_ 0.6) and p19 (OD_600_ 0.2). Then, 16 h later, plants were inoculated with TBSV and CIRV sap preparations. Plant samples were harvested 2 and 3 dpi, respectively. To test the effect of RavK expression, *N*. *benthamiana* leaves were co-agroinfiltrated with pGD-FlagRavK (OD_600_ 0.6) and p19. The agro-infiltrated leaves were inoculated with TBSV 16 h later. Plant samples were harvested at 2 dpi. Total plant RNA was extracted and tombusvirus RNA accumulation was detected by northern blot with CNV, TBSV and CIRV ^32^P-labeled probes as described [90, 93].

### Testing the effect of nuclear re-targeting of Rpn11 on TBSV replication in *N*. *benthamiana*

*N*. *benthamiana* leaves were co-agroinfiltrated with pGD-EGFP vector, pGD-EGFP-Rpn8 (OD_600_ 0.7) or pGD-EGFP-NRS-Rpn8 (OD_600_ 0.7) together with pGD-CNV^20KStop^ (OD_600_ 0.2) and pGD-p19 (OD_600_ 0.2). Total RNA was extracted from infiltrated leaves 2.5 days post agroinfiltration and CNV gRNA levels were analyzed by northern blot [90, 93]. Images were quantified with ImageQuant software. The quantifications were analyzed in excel and standard error was calculated.

### Biomolecular fluorescence complementation (BiFC) in Rpn11 knockdown plants

Knockdown of Rpn11 expression via VIGS is described in S1 Text. Rpn11-silenced leaves were co-agroinfiltrated 8.5 days after VIGS. Plasmids pGD-T33-cYFP (OD_600_ 0.2), pGD-CNV-20K-stop and one of the following pGD-nYFP-Pgk1 (OD_600_ 0.2), pGD-nYFP-PK1 (OD_600_ 0.2), pGD-nYFP-Pdc1 (OD_600_ 0.2), pGD-nYFP-Adh1 (OD_600_ 0.2) were used for agroinfiltration. For the BiFC control, pGD-cYFP vector (OD_600_ 0.2) and one of the plasmids above were used. Plant cells were visualized in the confocal laser microscope 1.5 days after agroinfiltration [51].

### BiFC in plants transiently expressing RavK effector

N. *benthamiana* leaves were co-agroinfiltrated with BiFC plasmids pGD-p33-cYFP (OD_600_ 0.2), pGD-CNV-20K-stop (OD_600_ 0.2), pGD-RFP-SKL (OD_600_ 0.2), pGD-RavK (OD_600_ 0.6), pGD-p19 (OD_600_ 0.15) and one of the following pGD-nYFP-Pgk1 (OD_600_ 0.2) or pGD-nYFP-PK1 (OD_600_ 0.2). Plant samples were visualized 50 h after agroinfiltration. Additional BiFC experiments are described in S1 Text.

### Measurement of relative ATP levels within VROs in yeast and plants

Relative ATP levels in yeast and plant cells were visualized based on the ATeam^YEMK^-p92-based biosensor using a confocal microscope [21, 65]. Briefly, BY4741 yeasts were co-transformed with UpCM189-Tet-RavK, HpESC-Gal-p33/Gal-DI72, LpGAD-ADH-ATeam^YEMK^-p92 orUpCM189-Tet or HpESC-Gal-p33/Gal-DI72, LpGAD-ADH-ATeam^YEMK^-p92 (or LpGAD-ADH-ATeam^RK^-p92 as a control). The transformed yeasts were pre-grown in SC-ULH^−^ media supplemented with 2% raffinose at 23°C for 14 h, then transferred to SC-ULH^−^ media supplemented with 2% glucose at 23°C for 4 h.

In the case of CIRV, BY4741 yeasts were transformed with UpCM189-Tet-RavK, HpGBK-CUP1-p36/Gal-DI72, LpGAD-CUP1-ATeam^YEMK^-p95 or UpCM189-Tet, HpGBK-CUP1-p36/Gal-DI72, LpGAD-CUP1-ATeam^YEMK^-p95 (or LpGAD-CUP1-ATeam^RK^-p95 as a control). The transformed yeasts were pre-grown in SC-ULH^−^ media supplemented with 2% raffinose and 25μM of CuSO_4_ at 23°C for 14 h, then transferred to SC-ULH^−^ media supplemented with 2% glucose and 50 μM of CuSO_4_ at 23°C for 4 h.

BY4741, *act1-132*^*ts*^ and *cof1-8*^*ts*^ yeasts were transformed with HpESC-Gal-p33/Gal-DI72, LpGAD-ADH-ATeam^YEMK^-p92 or LpGAD-ADH-ATeam^RK^-p92 as a control. The transformed yeasts were pre-grown in SC-LH^−^ media supplemented with 2% glucose at 23°C for 14 h and then transferred to SC-LH^−^ supplemented with 2% glucose at 23°C or 32°C for 1 h and 3 h. Confocal FRET images were taken with Olympus microscope. FRET was measured using Olympus FLUOVIEW software and ImageJ software. Graphics were done using Prism6 Software [21].

To visualize ATP production within the VROs in plant cells, *N*. *benthamiana* leaves were co-agroinfiltrated with pGD-FlagRavK (OD_600_ 0.06), pGD-p33-ATeam^YEMK^(OD_600_ 0.02), pGD-p19 (OD_600_ 0.02) or pGD-p33-ATeam^YEMK^(OD_600_ 0.02), pGD-p19 (OD_600_ 0.02) or pGD-Flag (OD_600_ 0.06). The agro-infiltrated leaves were inoculated with TBSV 16 h later. The images were taken 52 h after agroinfiltration. For CIRV-based experiments, *N*. *benthamiana* plants were co-agroinfiltrated with pGD-FlagRavK (OD_600_ 0.06), pGD-p36-ATeam^YEMK^ (OD_600_ 0.02), pGD-p19 (OD_600_ 0.02), pGD-CIRV (OD_600_ 0.02) or pGD-Flag (OD_600_ 0.06), pGD-p36-ATeam^YEMK^ (OD_600_ 0.02), pGD-p19 (OD_600_ 0.02), pGD-CIRV (OD_600_ 0.02). The images were taken 52 h after agroinfiltration and analyzed as describe above [20, 21].

## Supporting information

S1 Text Supplementary materials and methods. (DOC)

S1 Fig Gene silencing of Rpn11 in *N*. *benthamiana*. VIGS-based knockdown of Rpn11 in N. benthamiana. (A) Top Images: phenotypes of Rpn11 knockdown plants. Semi-quantitative RT-PCR shows the Rpn11 mRNA level after VIGS treatment. RT-PCR of tubulin mRNA and ribosomal RNA from the same samples are used as loading controls. (B) Western blot analysis of the ectopically-expressed His_6_-tagged glycolytic and fermentation enzymes in Rpn11 knockdown versus control VIGS (TRV-MBP-5’) plants. Total proteins in SDS-PAGE were stained with coomassie blue as controls.
(TIF)

S2 Fig Demonstration of sequestration of Rpn11 from the cytosol to the nucleus. (A) RFP-H2B transgenic *N*. *benthamiana* plants expressing GFP-NRS-Rpn8 were analyzed via confocal laser microscopy 2.5 days post-agroinfiltration. Control experiments included plants expressing GFP-Rpn8. (B) Expression of GFP-NRS-Rpn8 did not change the localization of TBSV p33-BFP replication protein. Control experiments show the partial re-localization of GFP-Rpn8 into p33-BFP foci (pointed by arrows). (C) Expression of GFP-NRS-Rpn8 sequesters RFP-Rpn11 into the nucleus. Bottom image: co-localization of GFP-Rpn8 and RFP-Rpn11 in the cytosol and the nucleus. Scale bar is 10 μm. Each experiment was repeated.
(TIF)

S3 Fig Temperature-sensitive mutation in Rpn11 reduces the co-purification of Tdh3 and Tdh2 glycolytic enzymes with the viral replicase. (A) Flag-p33 and Flag-p92 replication proteins were expressed in WT and *rpn11-14*^*ts*^ yeasts together with His_6_-Tdh3. First panel: Western blot analysis of co-purified His_6_-Tdh3 with TBSV replicase from detergent-solubilized membrane fraction of yeast cultured at either permissive (23°C) or semi-permissive (32°C) temperatures. The co-purified His_6_-Tdh3 was detected by western blot with anti-His antibody. Second panel: Western blot shows Flag-affinity purified p33 in the same samples as above with anti-Flag antibody. Third panel: Western blot analysis shows the levels of Flag-p33 in total protein extracts detected with anti-Flag antibody. Fourth panel: Western blots of His_6_-Tdh3 and His_6_-p33 in total protein extracts detected with anti-His antibody. Fifth panel: Coomassie-blue stained gel SDS gel of the total protein extracts as loading controls. (B) Flag-p33 and Flag-p92 replication proteins were expressed in WT and *rpn11-14*^*ts*^ yeasts together with His_6_-Tdh2 or His_6_-RH2 helicase. See further details in panel A.
(TIF)

S4 Fig Transient expression of *Legionella* VipA effector affects the architecture of the actin network and TBSV VROs in GFP-mTalin *N*. *benthamiana* transgenic plants. **(**A) Top row: Transgenic *N*. *benthamiana* plants expressing GFP-mTalin actin-binding protein, and co-expressing VipA, p33-BFP and RFP-SKL peroxisomal luminal marker (to visualize TBSV VROs). Second row: Control GFP-mTalin *N*. *benthamiana* plants expressing p33-BFP, RFP-SKL were visualized via confocal microscopy. The plants were infected with TBSV 16 h after agroinfiltration. Plant samples were analyzed using confocal microscopy 36 h post-infection. (B) The same experiment as in panel A, except plants did not express viral components. The plants were mock-inoculated. See details in panel A. (C) Transgenic *N*. *benthamiana* plants expressing GFP-mTalin, and co-expressing VipA, CIRV p36-BFP and RFP-Tim21 mitochondrial marker (to visualize CIRV VROs). The plants were also agroinfiltrated with pGD-CIRV. Plant samples were analyzed using confocal microscopy 36 h post-infection. Note that we show two sets of images to illustrate the enlarged size of CIRV VROs when VipA is co-expressed in plants. The control image is shown in Fig 7E, top image panel. (D) The same experiment as in panel C, except plants did not express viral components. The plants were mock-inoculated. See details in panel C. The scale bar is 10 μm. Each experiment was repeated.
(TIF)

S5 Fig Transient expression of RavK in *N*. *benthamiana*. (A-B) Lack of visible phenotypes of transient RavK expression on the leaves of *N*. *benthamiana* 4 d post-agroinfiltration. The leaves were inoculated with CNV or CIRV or mock-inoculated (C) Western blot analysis of the ectopically-expressed His_6_-tagged glycolytic Pgk1, His_6_-PK, His_6_-Fab2 and fermentation His_6_-Pdc1 and His_6_-Adh1 enzymes in RavK (detected via anti-Flag- rabbit antibody) expressing versus control *N*. *benthamiana* plants. Asterisk depicts a nonspecific band detected by the anti-Flag- rabbit antibody. Total proteins in SDS-PAGE were stained with Coomassie blue as controls.
(TIF)

S6 Fig Dependence of ATP generation within tombusvirus VROs on actin and cofilin in yeast. Relative ATP levels produced within the tombusvirus VROs was visualized via expressing ATeam-p92 in WT, act1^ts^ and cof1^ts^ yeasts at the permissive and semi-permissive (32°C) temperatures. The quantitative FRET values of multiple cells are shown in Fig 13B.
(TIF)
