# Supplementary material for: A novel viral strategy for host factor recruitment: The co-opted proteasomal Rpn11 protein interaction hub in cooperation with subverted actin filaments are targeted to deliver cytosolic host factors for viral replication
Source: PLoS Pathog. 2021 Jun 23;17(6):e1009680. doi: 10.1371/journal.ppat.1009680 (PMC8260003; doi:10.1371/journal.ppat.1009680)
Supplement: S1 Text — (DOC) [file ppat.1009680.s001.doc]

**Supplementary material**

**S1 Text. Supplementary materials and methods**

**Yeast expression plasmids.** The following yeast expression plasmids have been previously described: LpGAD-CUP1-Flag-p92, HpGBK-Cup1-Flag-p33/Gal-DI72, LpGAD-CUP1-His-p92, HpGBK-CUP1-His-p33/Gal-DI72 [1]. LpGAD‑ ADH-ATeam^YEMK^-p92, LpGAD-ADH-ATeam^RK^-p92, HpESC-CUP1-ATeam^YEMK^p95, HpESC-CUP1- ATeam^RK^-p95 [2], UpRS316-Tef-Pgk1 [3], UpYES-Cdc19 [2], UpYES-Pdc1, UpYES-Adh1 [4], UpYC‑Rpn11 [5], pCM189-Tdh3 [6], pYC2-Fba2 [7].

The following plant expression plasmids have been described before: pGD‑p33-ATeam^YEMK^, pGD-p36-ATeam^YEMK^ [2], pGD-p33-BFP, pGD‑p33RFP, pGD-nYFP-MBP, pGD-p33-cYFP and pGD-p36-cYFP [8]; pGD-MS2CP-RFP, pYC‑DI-72(-)-MS2 [9], pGD-nYFP-Pgk1, pGD-nYFP-PK [3], pGD‑nYFP-Pdc1, pGD‑nYFP-Adh1 [4], and pGD-nYFP-Fba2 [7].

To make the yeast expression plasmids pYC-NT-VipA and pYC-NT-VipA-N, the VipA gene sequence and VipA-N-terminal fragment were PCR-amplified from lpg0390 plasmid using the *Legionella pneumophila* effector library [10] with oligos #7434 - #7435 and #7434 - #7437, respectively. The PCR products were digested with BamH and XhoI restriction enzymes and inserted into UpYC2 vector previously digested with BamHI and XhoI [11]. To make plasmid pYC-RavK, the RavK gene sequence was PCR-amplified from lpg0969 plasmid using the *Legionella pneumophila* effector library [10] using primers #7664 - #7665. Then, the PCR products were digested with BamHI and XhoI and inserted into UpYC2 vector [11]. *Legionella* effector clones in pDONR223 were obtained from Dr. Craig R. Roy and B. Lindenbach (Yale University). Yeast plasmids pAG416GAL-ccdB-VipA and pAG416GAL-ccdB RavK were done by Jannine Baker using the Gateway cloning system [12].

To generate UpCM189-Tet-VipA and UpCM189-Tet-RavK plasmids, the VipA and RavK gene sequences were PCR-amplified with primers #7434 -#7435 and #7664 - #7665, respectively. The PCR products were digested with BglII and PstI restriction enzymes and cloned into BamHI-PstI digested-UpCM189 vector [13]. To construct pRS315-Gal-CycT vector, GalL promoter sequence from pFA6-pYM-N26 (Euroscarf) [14] was digested with SacI and SpeI restriction enzymes and PCR-amplified. The Cyc terminator sequence was PCR-amplified with primers #3728 - #3730. Then we cloned the GalL promoter sequence and CyCt sequence into pRS315 vector [15]. 3x HA-VipA and His_6_-RavK sequences were PCR-amplified with primers #7627 - #7435 and #1402 - #7665. The PCR products were digested with BamHI-SalI and BglII-XhoI restrictions enzymes, respectively, and cloned into BamHI-SalI-digested pRS315-Gal-CycT vector to obtain pRS315-Gal-HAVipA and pRS315-Gal-HisRavK plasmids, respectively.

To generate the plant expression plasmids: pGD-FlagVipA, pGD-2X35S-L-RFPVipA, pGD‑FlagRavK and pGD-2X35S-L-RFPRavK. The VipA gene sequence was PCR-amplified from lpg0390 plasmid and the RavK gene sequence was PCR-amplified from lpg0969 plasmid [10] using the primers #7434 - #7435 and #7664 - #7665, respectively. The PCR products were digested with BamHI and XhoI, and then inserted into BamHI-SalI digested pGD-Flag or pGD-RFP vectors. Plasmid pEarleyGate100-VipA was provided by Dr. Junichi Inaba [12]. To make plasmids pGD-2X35S-L-RFP-Pgk1 and pGD-2X35S-L-RFP-Fba2, *A. thaliana* cDNA preparation was used as a template to PCR-amplify the *PGK1* gene sequence with oligos #6437 and #6438 an the *FBA2* gene with oligos #8047 and #8048. The PCR products were digested with BclI and XhoI or BamHI and SalI restriction enzymes and cloned into pGD‑2X35S‑L‑NRFP plasmid [16] digested with BamHI and SalI.

To construct plasmids pGD-2x35S-L-RFP-Rpn11, pGD-2x35S-L-BFP-Rpn11, the *RPN11* gene sequence was PCR‑amplified from *A. thaliana* cDNA library with oligos #5984 - #5986 and cloned into digested BamHI and SalI plasmids pGD-2X35S-L-NRFP or pGD-2X35S-L-NBFP. To construct pTRV2-nMBP, the N-terminal region of the *MBP* gene sequence was PCR-amplified with oligos #6188 - #8336. The PCR product was digested with BamHI and XhoI restriction enzymes and inserted into pTRV2 VIGS plasmid [17], previously digested with BamHI-XhoI.

To build plasmids pGD‑NRS‑EGFP‑Rpn8, the nuclear retention signal (NRS) was PCR-amplified with oligos #6889 - #6876 from plasmid pCiNeo-3XFlag-NRS-NCL (Dr. Britt Glaunsinger, UC Berkeley) [9]. The eGFP fragment sequence was PCR-amplified with oligos #1292 - #4540, then, both PCR-products were digested with HindIII restriction enzyme and ligated together. The ligation product was then used as a template to PCR-amplify NRS-EGFP with oligos #6889 - #4540, followed by digestion of the PCR product with XhoI restriction enzyme. The *RPN8* gene sequence was PCR-amplified from *A. thaliana* cDNA library with oligos #8286 - #8287, then, the PCR product was digested with XhoI enzyme and ligated with XhoI digested-NRS‑EGFP. The final product NRS-EGFP-Rpn8 was PCR-amplified with oligos #6889 - #8287 and digested with BamHI restriction enzyme and inserted into pGD-2X35S-L-cflag previously digested with BamHI [8]. Note the final product has a stop codon, so the C-terminal Flag-tag is not translated. To construct plasmids pGD-EGFP-Rpn8, EGFP-Rpn8 fragment was PCR-amplified with oligos #2413 - #8287 digested with XbaI and BamHI restriction enzymes and cloned into pGD-cFlag digested with XbaI and BamHI. All the plasmid sequences were verified with DNA sequencing.

**VIGS-based knockdown of Rpn11 expression in *N. benthamiana* plants.** Knockdown of Rpn11 expression was performed via virus-induced gene silencing (VIGS) in *N. benthamiana* as before [18,19]. *N. benthamiana* plants were co-agroinfiltrated with pTRV1 (OD_600_ 0.05) and pTRV2-Rpn11 (OD_600_ 0.05) [5]. The pTRV2-nMBP (OD_600_ 0.05) construct was used as a VIGS control. To confirm Rpn11 silencing, we performed a semi-quantitative RT-PCR with primer oligo-d(T) for RT and primers #5698 - #5699 to PCR-amplify Rpn11 mRNA. Tubulin mRNA was used as an internal control and PCR-amplified with primers #2859 - #2860. Total protein was extracted from agroinfiltrated leaves (Molho et. al 2021b) and the protein accumulation of Pgk1, PK1, Pdc1 and Adh1 in Rpn11-silenced leaves or control leaves was analyzed by western blot. Proteins were detected with anti-FLAG antibody. Note Rpn11-silencing can have a strong phenotype in the plants, therefore, the plants were monitored daily to choose the best time for the silencing and BiFC analysis. Plant pictures were taken 10 days after VIGS treatment.

**BiFC in plants transiently expressing RavK effector.** For TBSV-based experiments, N*. benthamiana* leaves were co-agroinfiltrated with BiFC plasmids pGD-p33-cYFP (OD_600_ 0.2), pGD-RFP-SKL (OD_600_ 0.2), pGD-RavK (OD_600_ 0.6), pGD-p19 (OD_600_ 0.15) and pGD-nYFP-Pgk1 (OD_600_ 0.2) or pGD-nYFP-PK1 (OD_600_ 0.2). The agroinfiltrated plant leaves were inoculated with TBSV 16 h after agroinfiltration. Plant samples were visualized 36 h after infection via confocal laser microscopy.

For CIRV-based experiments, plants were co-agroinfiltrated with BiFC plasmids pGD-p36-cYFP (OD_600_ 0.2), pGD-Tim (OD_600_ 0.2), pGD-CIRV (OD_600_ 0.2), pGD-RavK (OD_600_ 0.6), pGD-p19 (OD_600_ 0.15) and one of the following: pGD-nYFP-Pgk1 (OD_600_ 0.2) or pGD-nYFP-PK1 (OD_600_ 0.2). Plant samples were visualized 50 h after agroinfiltration via confocal laser microscopy. As a control, instead of pGD-RavK, plant leaves were agroinfiltrated with pGD vector (OD_600_ 0.6). As a BiFC negative control, plants were co-agroinfiltrated with pGD-cYFP (OD_600_ 0.2), pGD-RFP-SKL (OD_600_ 0.2), pGD-RavK (OD_600_ 0.6) and pGD-p19 (OD_600_ 0.15). Total plant protein was extracted as previously (Molho et. al 2021b) and analyzed by western blot [20]. Pgk1 and PK proteins were detected using anti-FLAG antibody.

**Confocal laser microscopy based on transgenic GFP-mTalin *N. benthamiana*.** To observe the effect of VipA and RavK effectors on the plant actin network, we used transgenic *N. benthamiana* expressing GFP-mTalin, which binds selectively to actin filaments. The plants were co-agroinfiltrated with pGD-p33-BFP (OD_600_ 0.2), pGD-RFP-SKL (OD_600_ 0.2), pGD-2x35SL-FlagVipA (OD_600_ 0.6) and pGD-p19 (OD_600_ 0.2) or pGD-p33-BFP (OD_600_ 0.2), pGD-RFP-SKL (OD_600_ 0.2), pGD-2x35SL-FlagRavK (OD_600_ 0.6) and pGD-p19 (OD_600_ 0.2). Agroinfiltrated plant leaves were inoculated with TBSV 16 h after agroinfiltration. Confocal laser microscopy images were taken with an Olympus FV1000 or FV3000 microscopes (Olympus America) 36 h after infection. As a control, plant leaves were agroinfiltrated with pGD-p33-BFP (OD_600_ 0.2), pGD-RFP-SKL (OD_600_ 0.2) and pGD-2x35SL- (OD_600_ 0.6). Plant leaves were infiltrated with pGD-RFP-SKL (OD_600_ 0.2), pGD-2x35SL-FlagVipA (OD_600_ 0.6) and pGD-p19 (OD_600_ 0.2) or pGD-RFP-SKL (OD_600_ 0.2), pGD-2x35SL-FlagRavK (OD_600_ 0.6) and pGD-p19 (OD_600_ 0.2). Plant cells were analyzed 52 h after agroinfiltration via confocal laser microscopy.

For CIRV-based studies, N*. benthamiana* plants were co-agroinfiltrated with pGD-p36-BFP (OD_600_ 0.2), pGD-RFP-Tim21 (OD_600_ 0.2), pGD-2x35SL-FlagVipA (OD_600_ 0.6), pGD-p19 (OD_600_ 0.2) and pGD-CIRV (OD_600_ 0.2) or pGD-p36-BFP (OD_600_0.2), pGD-RFP-Tim21 (OD_600_ 0.2), pGD-2x35SL-FlagRavK (OD_600_ 0.6), pGD-p19 (OD_600_ 0.2) and pGD-CIRV (OD_600_ 0.2). As a control, plant leaves were agroinfiltrated with pGD-p36-BFP (OD_600_ 0.2), pGD-RFP-Tim21 (OD_600_ 0.2), pGD-2x35SL (OD_600_ 0.6) and pGD-CIRV (OD_600_ 0.2). Plant leaves were infiltrated with pGD-RFP-Tim21 (OD_600_ 0.2), pGD-2x35SL-FlagVipA (OD_600_ 0.6) and pGD-p19 (OD_600_ 0.2) or pGD-RFP-Tim21 (OD_600_ 0.2), pGD-2x35SL-FlagRavK (OD_600_ 0.6) and pGD-p19 (OD_600_ 0.2). Plant cells were analyzed 52 h later in a confocal laser scanning microscope.

To observe the subcellular localization of Rpn11 and the actin filaments in the presence or absence of viral components in plant cells, transgenic *N. benthamiana* expressing GFP-mTalin plants were co-agroinfiltrated with pGD-p33-RFP (OD_600_ 0.2), pGD-BFP-Rpn11 (OD_600_ 0.3), pGD-p19 (OD_600_ 0.2). Agroinfiltrated plant leaves were inoculated with TBSV 16 h later. Transgenic *N. benthamiana* expressing GFP-mTalin plants were co-agroinfiltrated with pGD-BFP-Rpn11 (OD_600_ 0.3), pGD-p19 (OD_600_ 0.2). Plant cells were visualized 52 h after agroinfiltration with a confocal microscope [8].

**Confocal microscopy in plants.** To test the role of Rpn11 in the recruitment of other pro-viral host factors, *N. benthamiana* leaves were co-agroinfiltrated with pGD-NRS-EGFP-Rpn8 (OD_600_ 0.7), pGD-p33-BFP (OD_600_ 0.2), pGD‑RFP‑Pgk1 (OD_600_ 0.2), pGD-19 (OD_600_ 0.15), pGD-CNV-20kstop (OD_600_ 0.2) or pGD‑EGFP-Rpn8 (OD_600_ 0.7), pGD-p33-BFP (OD_600_ 0.2), pGD-RFP-Pgk1 (OD_600_ 0.2), pGD-19 (OD_600_ 0.15), pGD-CNV-20kstop (OD_600_ 0.2) or pGD vector (OD_600_ 0.7), pGD-p33-BFP (OD_600_ 0.2), pGD-RFP-Pgk1 (OD_600_ 0.2), pGD-19 (OD_600_ 0.15), pGD-CNV-20kstop (OD_600_ 0.2). As control, plant leaves were co-agroinfiltrated with the following plasmids: pGD-NRS-EGFP-Rpn8 (OD_600_ 0.7), pGD-RFP-Pgk1 (OD_600_ 0.2) and pGD-19 (OD_600_ 0.15) or pGD-EGFP-Rpn8 (OD_600_ 0.7), pGD-RFP-Pgk1 (OD_600_ 0.2) and pGD-19 (OD_600_ 0.15) or pGD vector, pGD-RFP-Pgk1 (OD_600_ 0.2) and pGD-19 (OD_600_ 0.15). Plant cell images were taken using confocal microscope 48 h time-point after agroinfiltration. Similar experiments were performed for Adh1 and Pdc1 fermentation proteins, using plasmids pGD-RFP-Adh1 (OD_600_ 0.2) and pGD-RFP-Pdc1 (OD_600_ 0.2) for agroinfiltration [4].

**References**

1. Barajas D, Jiang Y, Nagy PD (2009) A unique role for the host ESCRT proteins in replication of Tomato bushy stunt virus. PLoS Pathog 5: e1000705.

**2. Chuang C, Prasanth KR, Nagy PD (2017) The Glycolytic Pyruvate Kinase Is Recruited Directly into the Viral Replicase Complex to Generate ATP for RNA Synthesis. Cell Host Microbe 22: 639-652 e637.**

**3. Prasanth KR, Chuang C, Nagy PD (2017) Co-opting ATP-generating glycolytic enzyme PGK1 phosphoglycerate kinase facilitates the assembly of viral replicase complexes. PLoS Pathog 13: e1006689.**

**4. Lin W, Liu Y, Molho M, Zhang S, Wang L, et al. (2019) Co-opting the fermentation pathway for tombusvirus replication: Compartmentalization of cellular metabolic pathways for rapid ATP generation. PLoS Pathog 15: e1008092.**

**5. Prasanth KR, Barajas D, Nagy PD (2015) The Proteasomal Rpn11 Metalloprotease Suppresses Tombusvirus RNA Recombination and Promotes Viral Replication via Facilitating Assembly of the Viral Replicase Complex. Journal of Virology 89: 2750-2763.**

**6. Wang RYL, Nagy PD (2008) Tomato bushy stunt virus co-opts the RNA-binding function of a host metabolic enzyme for viral genomic RNA synthesis. Cell Host & Microbe 3: 178-187.**

**7. Molho M, Chuang C, Nagy PD (2021) Co-opting of nonATP-generating glycolytic enzymes for TBSV replication. Virology 559: 15-29.**

**8. Xu K, Nagy PD (2016) Enrichment of Phosphatidylethanolamine in Viral Replication Compartments via Co-opting the Endosomal Rab5 Small GTPase by a Positive-Strand RNA Virus. PLoS Biol 14: e2000128.**

**9. Wu CY, Nagy PD (2019) Blocking tombusvirus replication through the antiviral functions of DDX17-like RH30 DEAD-box helicase. PLoS Pathog 15: e1007771.**

**10. Shames SR, Liu L, Havey JC, Schofield WB, Goodman AL, et al. (2017) Multiple Legionella pneumophila effector virulence phenotypes revealed through high-throughput analysis of targeted mutant libraries. Proc Natl Acad Sci U S A 114: E10446-E10454.**

**11. Barajas D, Li ZH, Nagy PD (2009) The Nedd4-Type Rsp5p Ubiquitin Ligase Inhibits Tombusvirus Replication by Regulating Degradation of the p92 Replication Protein and Decreasing the Activity of the Tombusvirus Replicase. Journal of Virology 83: 11751-11764.**

**12. Inaba JI, Xu K, Kovalev N, Ramanathan H, Roy CR, et al. (2019) Screening Legionella effectors for antiviral effects reveals Rab1 GTPase as a proviral factor coopted for tombusvirus replication. Proc Natl Acad Sci U S A 116: 21739-21747.**

**13. Gari E, Piedrafita L, Aldea M, Herrero E (1997) A Set of Vectors with a Tetracycline Regulatable Promoter System for Modulated Gene Expression in Saccharomyces cerevisiae. Yeast 13: 837-848.**

**14. Janke C, Magiera MM, Rathfelder N, Taxis C, Reber S, et al. (2004) A versatile toolbox for PCR-based tagging of yeast genes: new fluorescent proteins, more markers and promoter substitution cassettes. Yeast 21: 947-962.**

**15. Sikorski R, Hieter P (1989) A System of Shuttle Vectors and Yeast Host Strains Designed for Efficient Manipulation of DNA in Saccharomyces ceratisiae. Genetics: 19-27.**

**16. Xu K, Lin JY, Nagy PD (2014) The Hop-Like Stress-Induced Protein 1 Cochaperone Is a Novel Cell-Intrinsic Restriction Factor for Mitochondrial Tombusvirus Replication. Journal of Virology 88: 9361-9378.**

**17. Dinesh-Kumar SP, Anandalakshmi R, Marathe R, Schiff M, Liu Y (2003) Virus-induced gene silencing. Methods Mol Biol 236: 287-294.**

**18. Jaag HM, Nagy PD (2009) Silencing of Nicotiana benthamiana Xrn4p exoribonuclease promotes tombusvirus RNA accumulation and recombination. Virology 386: 344-352.**

**19. Dinesh-Kumar SP, Anandalakshmi R, Marathe R, Schiff M, Liu Y (2003) Virus-Induced Gene Silencing. In: Grotewold E, editor. Plant Functional Genomics. Totowa, NJ: Humana Press. pp. 287-293.**

**20. Panaviene Z, Panavas T, Serva S, Nagy PD (2004) Purification of the Cucumber necrosis virus replicase from yeast cells: Role of coexpressed viral RNA in stimulation of replicase activity. Journal of Virology 78: 8254-8263.**
