# Supplementary figures and images for: A novel viral strategy for host factor recruitment: The co-opted proteasomal Rpn11 protein interaction hub in cooperation with subverted actin filaments are targeted to deliver cytosolic host factors for viral replication
Source: PLoS Pathog. 2021 Jun 23;17(6):e1009680. doi: 10.1371/journal.ppat.1009680 (PMC8260003; doi:10.1371/journal.ppat.1009680)

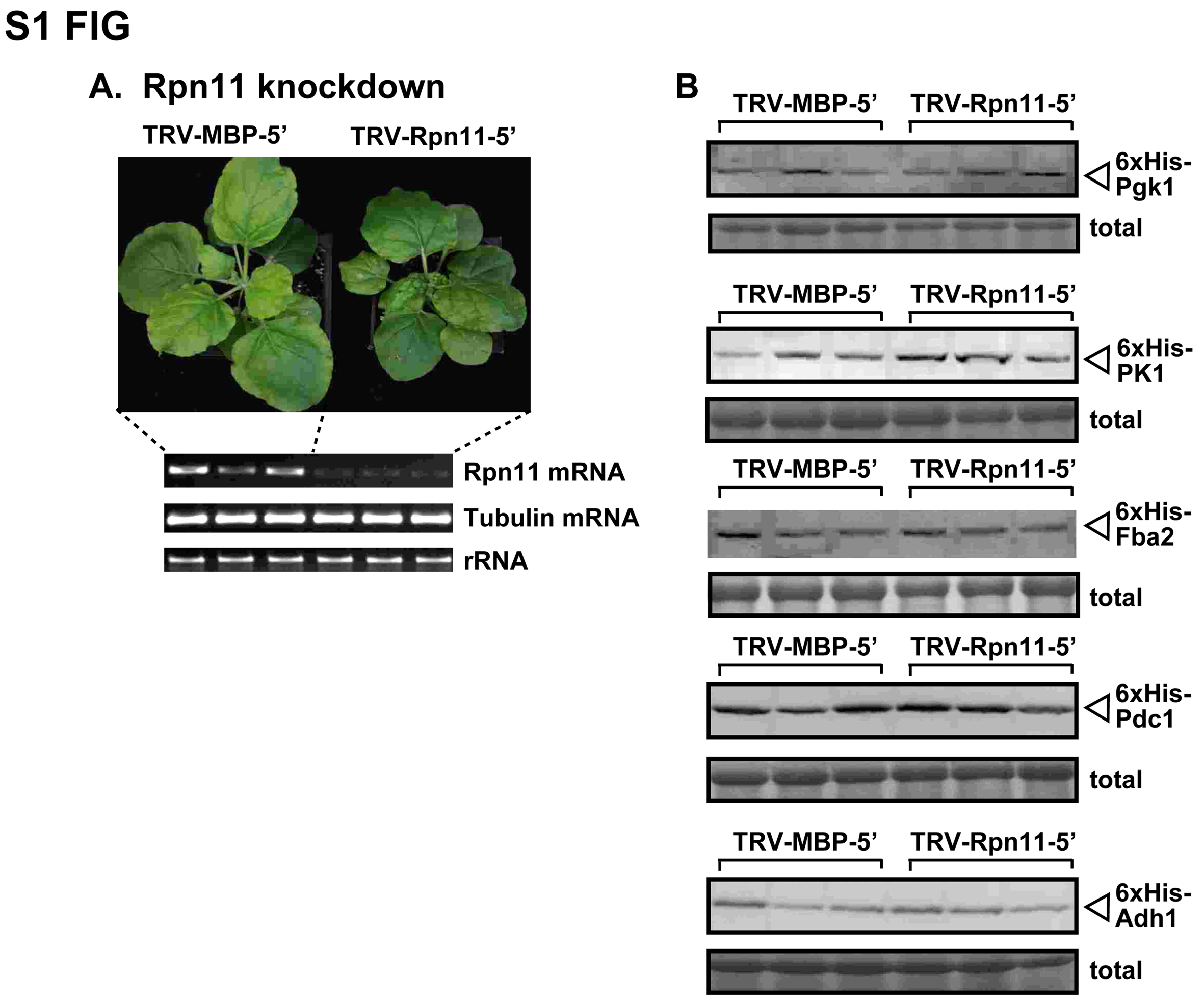

Supplement: S1 Fig — VIGS-based knockdown of Rpn11 in N. benthamiana. (A) Top Images: phenotypes of Rpn11 knockdown plants. Semi-quantitative RT-PCR shows the Rpn11 mRNA level after VIGS treatment. RT-PCR of tubulin mRNA and ribosomal RNA from the same samples are used as loading controls. (B) Western blot analysis of the ectopically-expressed His6-tagged glycolytic and fermentation enzymes in Rpn11 knockdown versus control VIGS (TRV-MBP-5’) plants. Total proteins in SDS-PAGE were stained with coomassie blue as controls. (TIF) [file ppat.1009680.s002.tif]

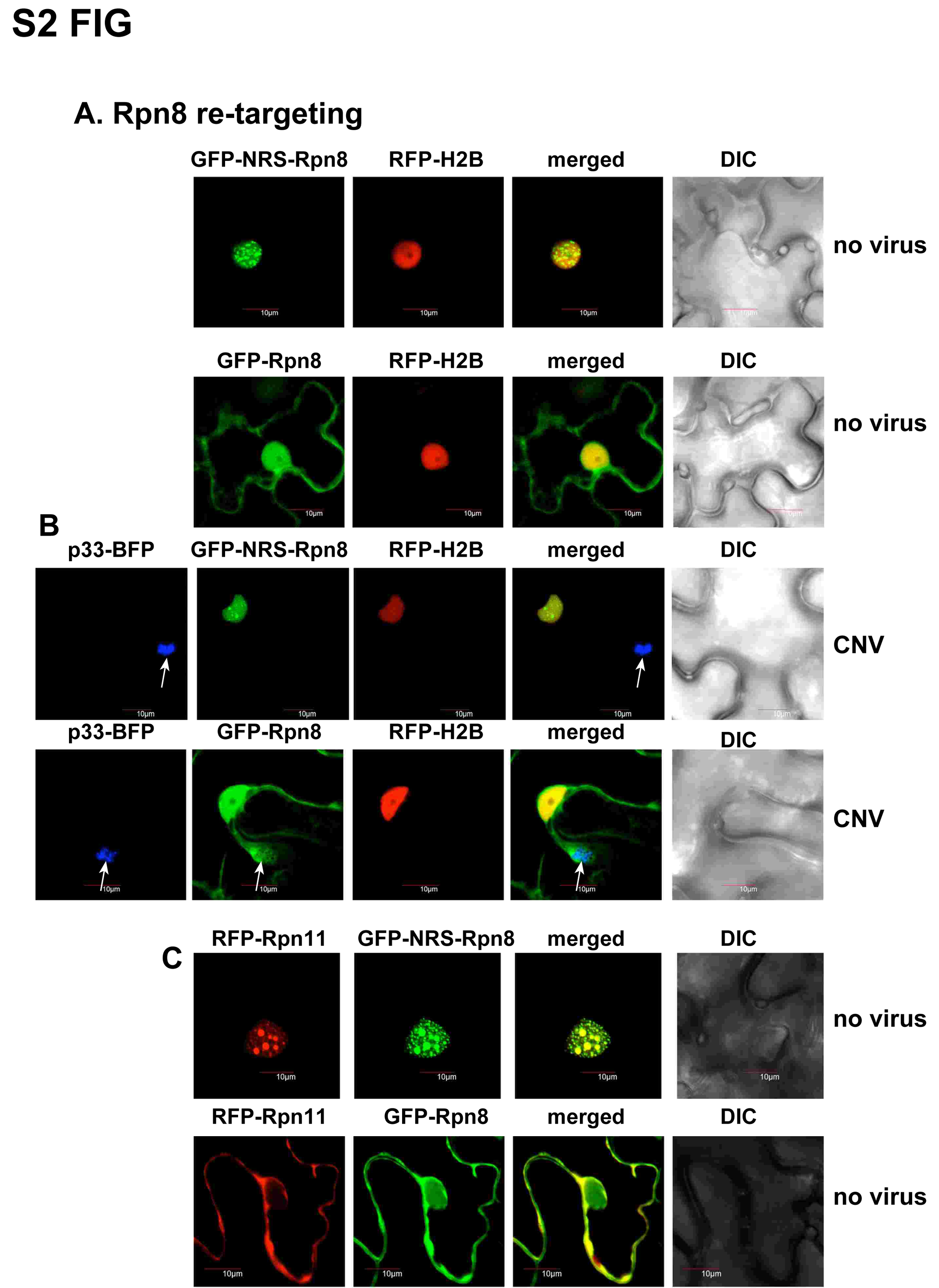

Supplement: S2 Fig — (A) RFP-H2B transgenic N. benthamiana plants expressing GFP-NRS-Rpn8 were analyzed via confocal laser microscopy 2.5 days post-agroinfiltration. Control experiments included plants expressing GFP-Rpn8. (B) Expression of GFP-NRS-Rpn8 did not change the localization of TBSV p33-BFP replication protein. Control experiments show the partial re-localization of GFP-Rpn8 into p33-BFP foci (pointed by arrows). (C) Expression of GFP-NRS-Rpn8 sequesters RFP-Rpn11 into the nucleus. Bottom image: co-localization of GFP-Rpn8 and RFP-Rpn11 in the cytosol and the nucleus. Scale bar is 10 μm. Each experiment was repeated. (TIF) [file ppat.1009680.s003.tif]

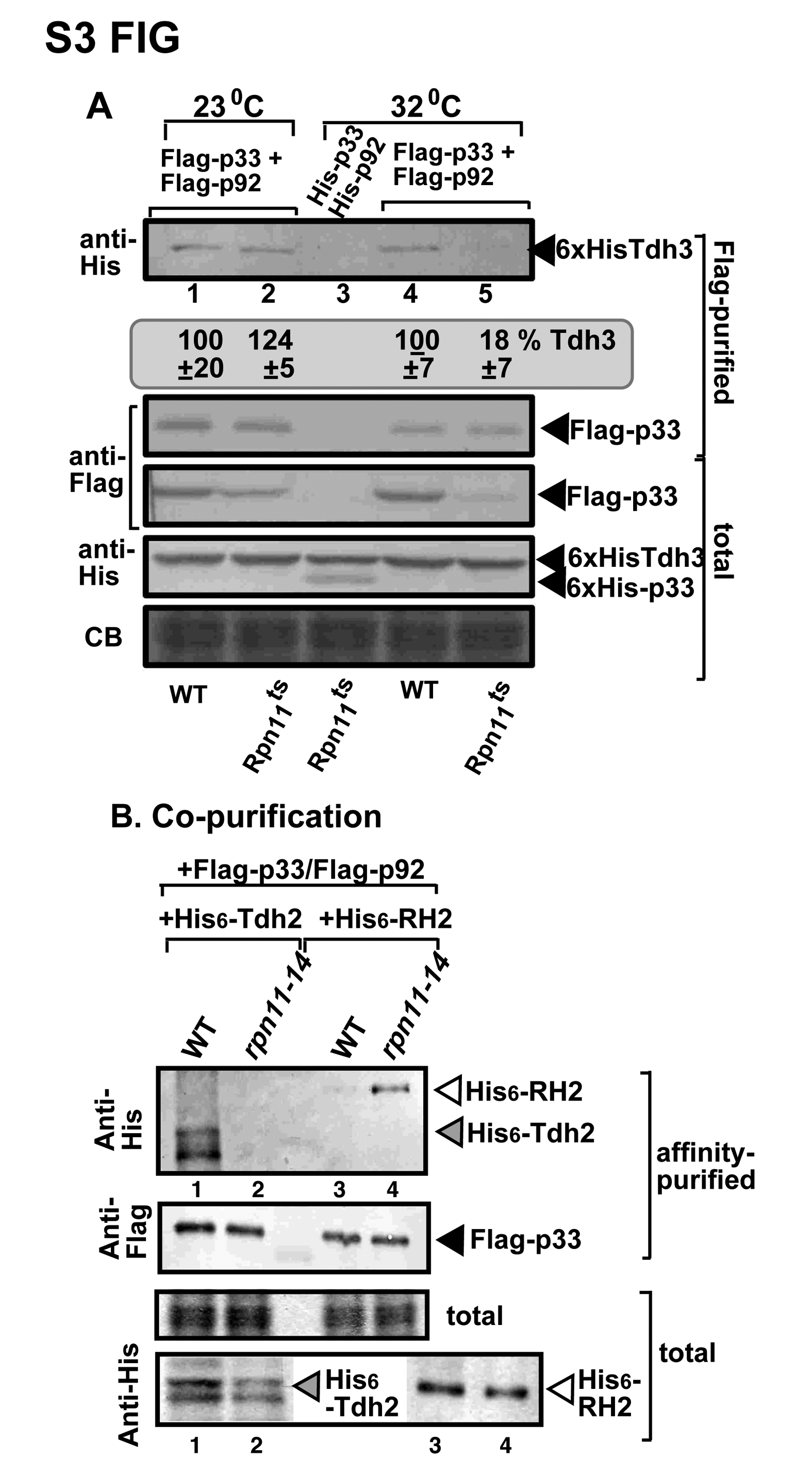

Supplement: S3 Fig — (A) Flag-p33 and Flag-p92 replication proteins were expressed in WT and rpn11-14ts yeasts together with His6-Tdh3. First panel: Western blot analysis of co-purified His6-Tdh3 with TBSV replicase from detergent-solubilized membrane fraction of yeast cultured at either permissive (23°C) or semi-permissive (32°C) temperatures. The co-purified His6-Tdh3 was detected by western blot with anti-His antibody. Second panel: Western blot shows Flag-affinity purified p33 in the same samples as above with anti-Flag antibody. Third panel: Western blot analysis shows the levels of Flag-p33 in total protein extracts detected with anti-Flag antibody. Fourth panel: Western blots of His6-Tdh3 and His6-p33 in total protein extracts detected with anti-His antibody. Fifth panel: Coomassie-blue stained gel SDS gel of the total protein extracts as loading controls. (B) Flag-p33 and Flag-p92 replication proteins were expressed in WT and rpn11-14ts yeasts together with His6-Tdh2 or His6-RH2 helicase. See further details in panel A. (TIF) [file ppat.1009680.s004.tif]

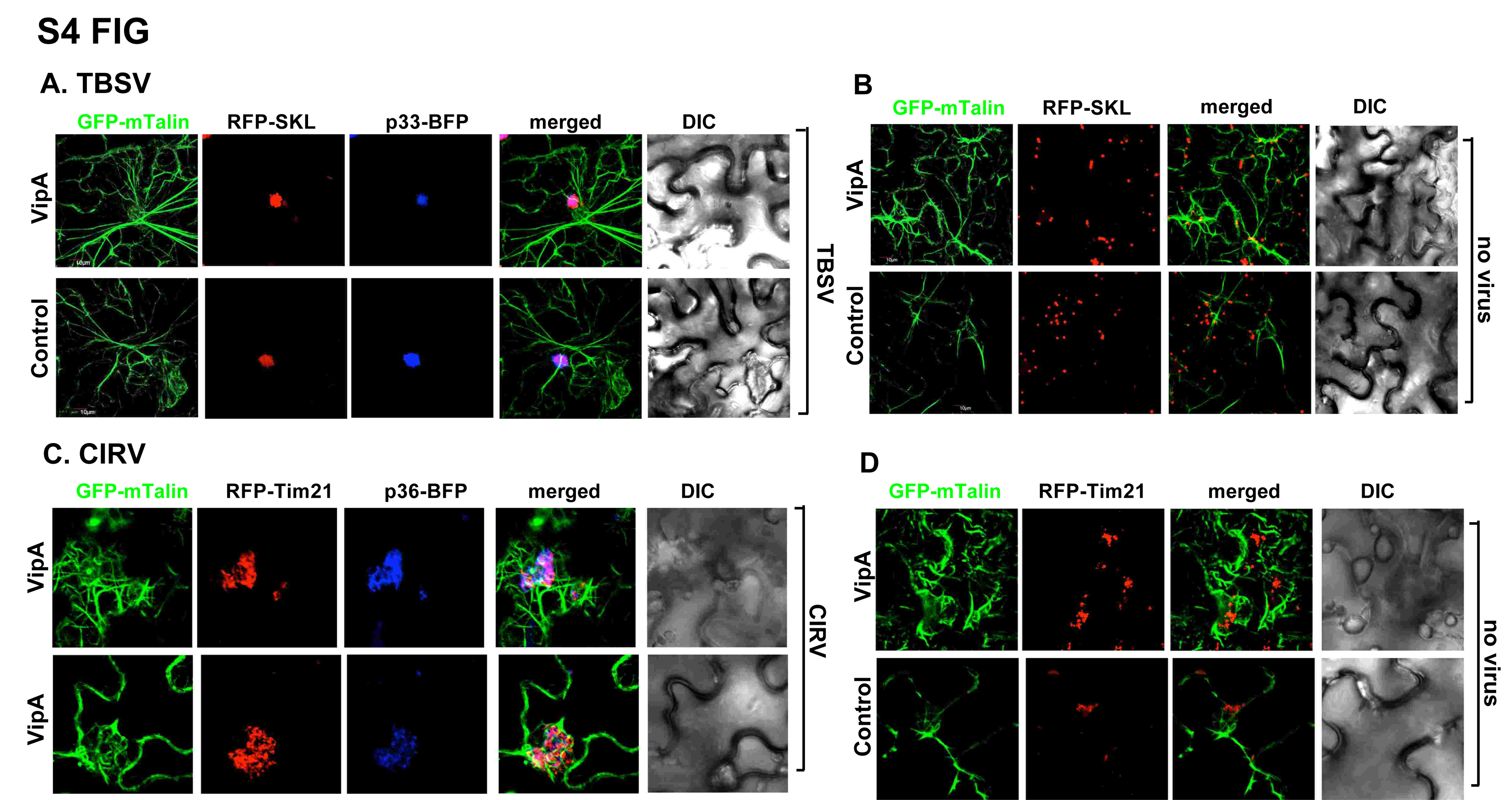

Supplement: S4 Fig — (A) Top row: Transgenic N. benthamiana plants expressing GFP-mTalin actin-binding protein, and co-expressing VipA, p33-BFP and RFP-SKL peroxisomal luminal marker (to visualize TBSV VROs). Second row: Control GFP-mTalin N. benthamiana plants expressing p33-BFP, RFP-SKL were visualized via confocal microscopy. The plants were infected with TBSV 16 h after agroinfiltration. Plant samples were analyzed using confocal microscopy 36 h post-infection. (B) The same experiment as in panel A, except plants did not express viral components. The plants were mock-inoculated. See details in panel A. (C) Transgenic N. benthamiana plants expressing GFP-mTalin, and co-expressing VipA, CIRV p36-BFP and RFP-Tim21 mitochondrial marker (to visualize CIRV VROs). The plants were also agroinfiltrated with pGD-CIRV. Plant samples were analyzed using confocal microscopy 36 h post-infection. Note that we show two sets of images to illustrate the enlarged size of CIRV VROs when VipA is co-expressed in plants. The control image is shown in Fig 7E, top image panel. (D) The same experiment as in panel C, except plants did not express viral components. The plants were mock-inoculated. See details in panel C. The scale bar is 10 μm. Each experiment was repeated. (TIF) [file ppat.1009680.s005.tif]

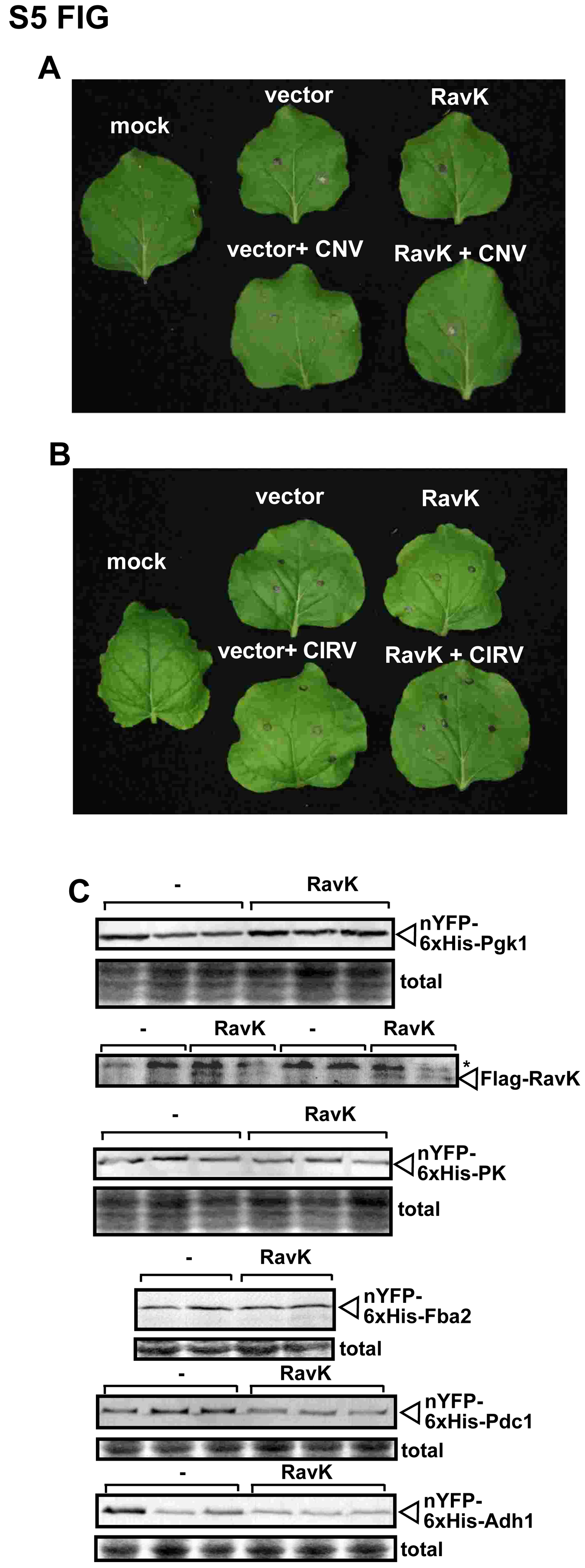

Supplement: S5 Fig — (A-B) Lack of visible phenotypes of transient RavK expression on the leaves of N. benthamiana 4 d post-agroinfiltration. The leaves were inoculated with CNV or CIRV or mock-inoculated (C) Western blot analysis of the ectopically-expressed His6-tagged glycolytic Pgk1, His6-PK, His6-Fab2 and fermentation His6-Pdc1 and His6-Adh1 enzymes in RavK (detected via anti-Flag- rabbit antibody) expressing versus control N. benthamiana plants. Asterisk depicts a nonspecific band detected by the anti-Flag- rabbit antibody. Total proteins in SDS-PAGE were stained with Coomassie blue as controls. (TIF) [file ppat.1009680.s006.tif]

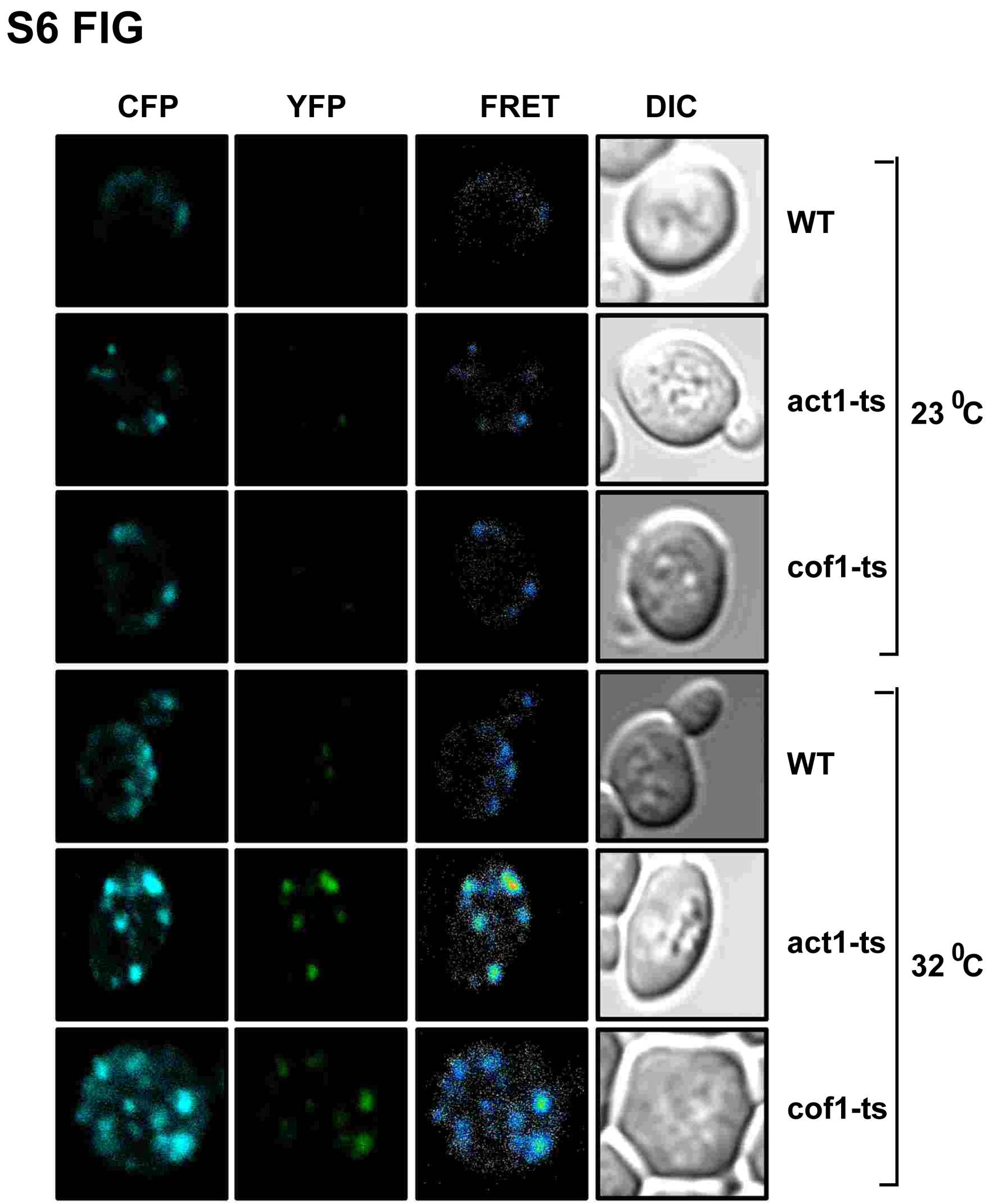

Supplement: S6 Fig — Relative ATP levels produced within the tombusvirus VROs was visualized via expressing ATeam-p92 in WT, act1ts and cof1ts yeasts at the permissive and semi-permissive (32°C) temperatures. The quantitative FRET values of multiple cells are shown in Fig 13B. (TIF) [file ppat.1009680.s007.tif]
